# QSAR Regression Models for Predicting HMG-CoA Reductase Inhibition

**DOI:** 10.3390/ph17111448

**Published:** 2024-10-30

**Authors:** Robert Ancuceanu, Patriciu Constantin Popovici, Doina Drăgănescu, Ștefan Busnatu, Beatrice Elena Lascu, Mihaela Dinu

**Affiliations:** 1 Department of Pharmaceutical Botany and Cell Biology, Faculty of Pharmacy, Carol Davila University of Medicine and Pharmacy, 020021 Bucharest, Romania; robert.ancuceanu@umfcd.ro (R.A.); patriciu.popovici@drd.umfcd.ro (P.C.P.); beatrice-elena.lascu@drd.umfcd.ro (B.E.L.); mihaela.dinu@umfcd.ro (M.D.); 2 Department of Pharmaceutical Physics, Faculty of Pharmacy, Carol Davila University of Medicine and Pharmacy, 020021 Bucharest, Romania; 3 Department of Cardiology, Carol Davila University of Medicine and Pharmacy, 020021 Bucharest, Romania; stefan.busnatu@umfcd.ro; 4 Emergency Hospital “Bagdasar-Arseni”, 050474 Bucharest, Romania

**Keywords:** HMG-CoA reductase, QSAR, statins, nested cross-validation, virtual screening, *Iris germanica*, machine learning, feature selection, mlr3, MACCS fingerprints, molecular descriptors

## Abstract

Background/Objectives: HMG-CoA reductase is an enzyme that regulates the initial stage of cholesterol synthesis, and its inhibitors are widely used in the treatment of cardiovascular diseases. Methods: We have created a set of quantitative structure-activity relationship (QSAR) models for human HMG-CoA reductase inhibitors using nested cross-validation as the primary validation method. To develop the QSAR models, we employed various machine learning regression algorithms, feature selection methods, and fingerprints or descriptor datasets. Results: We built and evaluated a total of 300 models, selecting 21 that demonstrated good performance (coefficient of determination, R^2^ ≥ 0.70 or concordance correlation coefficient, CCC ≥ 0.85). Six of these top-performing models met both performance criteria and were used to construct five ensemble models. We identified the descriptors most important in explaining HMG-CoA inhibition for each of the six best-performing models. We used the top models to search through over 220,000 chemical compounds from a large database (ZINC 15) for potential new inhibitors. Only a small fraction (237 out of approximately 220,000 compounds) had reliable predictions with mean pIC_50_ values ≥ 8 (IC_50_ values ≤ 10 nM). Our svm-based ensemble model predicted IC_50_ values < 10 nM for roughly 0.08% of the screened compounds. We have also illustrated the potential applications of these QSAR models in understanding the cholesterol-lowering activities of herbal extracts, such as those reported for an extract prepared from the *Iris × germanica* rhizome. Conclusions: Our QSAR models can accurately predict human HMG-CoA reductase inhibitors, having the potential to accelerate the discovery of novel cholesterol-lowering agents and may also be applied to understand the mechanisms underlying the reported cholesterol-lowering activities of herbal extracts.

## 1. Introduction

The incidence of atherosclerotic cardiovascular disease is on the rise and continues to rank as the top cause of death and disability in industrialized countries. Atherosclerosis can be slowed or even reversed with the use of lipid-lowering agents when the medicines are administered in appropriate regimens, while the plaque is still immature and has not become calcified or fibrotic [1]. Evidence from both primary and secondary prevention studies shows that HMG-CoA reductase inhibitors (also known as statins) lessen the risk of atherosclerotic cardiovascular disease, making them the first-line lipid-lowering agents recommended by various national and international clinical guidelines [2]. HMG-CoA reductase (3-hydroxy-3-methylglutaryl coenzyme A reductase) is an enzyme that catalyzes an initial stage in the biosynthesis of cholesterol. This particular step is the one controlling the overall speed of the entire sequence of reactions involved in cholesterol synthesis [3]. Besides their main effects on HMG-CoA reductase, such inhibitors appear to have a large number of pleiotropic effects, providing cardiovascular protection independent of their effect on cholesterol, by preventing the formation of intermediates in the cholesterol biosynthetic pathway. These effects result in an inhibition of post-translational modifications of intracellular proteins. These changes, in turn, have downstream effects on endothelial, inflammatory, and smooth muscle cells [4]. The pleiotropic effects of statins and their potential therapeutic uses (related to the cholesterol inhibition or their pleiotropic effects) seem to be broad, ranging from anti-inflammatory and immunomodulatory activities [5] to neuroprotective effects [6,7], from anti-tumorigenic and anti-metastatic actions [8,9] to protection against aging [10], from preventing or reducing the risk of osteoporosis [11] to certain effects on the endocrine system [12]. This topic, however, remains controversial, and the true impact of the reduction in these intermediates has not been fully clarified because it frequently corresponds to a simultaneous fall in cholesterol [4].

The currently available statins differ widely in their solubility and pharmacokinetic properties. Some are rather lipophilic (simvastatin, fluvastatin, lovastatin, pitavastatin, and atorvastatin), can easily penetrate biological membranes, and tend to be more widely distributed in the body. Others, like pravastatin and, to a lesser extent, rosuvastatin, are more hydrophilic. They stay connected to the polar surface of the membrane and need protein transporters to get into the cell. It is thought that because they are not as widely distributed, they might have fewer pleiotropic effects [13]. Whereas approved statins seem often to be similar in their efficacy and safety, there are data suggesting that different statins have different safety profiles (with respect to their muscle-related side effects [14], liver toxicity [15,16], diabetes-risk [17], Alzheimer disease risk [18], drug interactions, etc. [19]) and different efficacy [20]. Therefore, developing new HMG-CoA reductase inhibitors could result in statins with improved or modified efficacy and safety.

QSAR is a computational approach that is based on building models describing the relationship between the biological activity and certain structural properties (descriptors) of ligands that bind to a specific biologic target (or who have a specific biological effect) [21]. Over time, two primary approaches to QSAR have emerged, which differ based on the methods used to build the models. The first, more traditional, approach is based on models that are often straightforward, linear, and may be interpreted in terms of physicochemical concepts. The second approach is based on the utilization of machine learning techniques, which are more suited for predicting the relationship between structure and activity in extensive datasets with significant chemical variability [22]. Molecular descriptors can capture broad categories of molecule structure information, such as bulk characteristics, substructure frequency, or more complicated three-dimensional descriptions. To describe the level of complexity for such descriptors, different dimensionalities (levels of complexity) are used, the descriptors being labelled as 1D, 2D, 3D, and 4D [23]. While it is reasonable to assume that 3D models would provide substantially more detail regarding a compound’s activity or property, in practice, such models are typically restricted to relatively small series of similar compounds, in order to eliminate conformational uncertainty. On the other hand, 2D molecule representations are commonly used for large datasets. Furthermore, the molecular graphs provided by 2D representations are also useful for interpreting QSAR models via the use of chemical structure information (molecule fragments) [24].

Rajathei et al. (2020) developed a 2D-QSAR model for HMG-CoA reductase inhibitors, but it was based on only 30 pyrrole derivatives of atorvastatin [25]. Moorthy et al. (2015) developed an interesting set of QSAR models based on both linear regression and classification, using MOE for the calculation of the molecular descriptors (2D and 3D). However, these authors did not report on using the models for virtual screening purposes and their validation was based on the techniques of leave one out (LOO), leave many out (LMO), and bootstrapping (besides randomization and holdout testing) [26]. Nested cross-validation, which is able to provide a more reliable estimation of model performance and a better control of overfitting, was not used in this paper. Moreover, it is not clear from that paper whether the HMG-CoA reductase inhibitors were evaluated on a human or rodent version of the enzyme. Samizo and Kaneko (2023) developed QSAR models using a dataset of 833 compounds from the ChEMBL database, but they used a HMG-CoA reductase of rat origin, not of human origin [27]. Zang et al. (2017) built a 3D-QSAR model based on a small sample size of 19 compounds, but targeting lepidopteran, not human, HMG-CoA reductase [28]. Another QSAR model was also built on a small number (*n* = 18) of phthalimide congeners [29]. We report in this paper on a series of QSAR models developed for human HMG-CoA reductase inhibitors, using nested cross-validation as the main validation approach, and using the best performing-models for the virtual screening of over 220,000 chemical compounds from the ZINC 15 database. As a practical application of the models, we have also used them to understand which are the natural compounds responsible for the reported LDL-cholesterol lowering effect of an *Iris* × *germanica* L. extract [30].

## 2. Results

### 2.1. Chemical Space Distribution and Diversity of the Compounds in the Training Data Set

**Figure 1:**
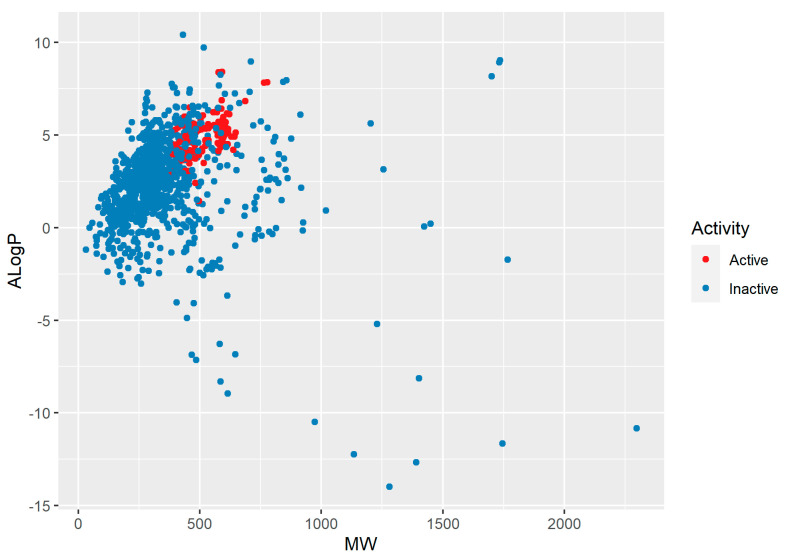
Chemical diversity representation of the HMGCoA-inhibitors dataset (chemical space defined by the molecular weight (MW) and AK Ghose-G.M. Crippen logP (ALogP).

The variation of ALogP (a measure of lipophilicity, and indirectly, of membrane permeability [31]) as a function of the molecular weight is represented graphically in Figure 1. The largest density was observed for molecular weights varying between 250 and 500 g mol^−1^ (first and third quartiles corresponded to 257.2 and 455.5, respectively), and for ALogP varying between 1 and 4. For active compounds (defined as having an IC_50_ < 100 nM), the minimum molecular weight in the dataset was 369.4, the maximum 778.1, and the median value was 491.1 g mol^−1^. ALogP varied between 1.4 and 8.4 for the active compounds, with a median value of 4.9. We compared these data with those for ten statins that were at least partially developed as medicinal products (lovastatin, cerivastatin, atorvastatin, fluvastatin, simvastatin, rosuvastatin, glenvastatin, pravastatin, mevastatin, and pitavastatin) and found that, for the latter, molecular weight varied between 390.5 and 558.6, with a median value of 422.5 g mol^−1^. For statins, ALogP ranged between 2.1 and 5.5, with a median value of 4.2.

**Figure 2:**
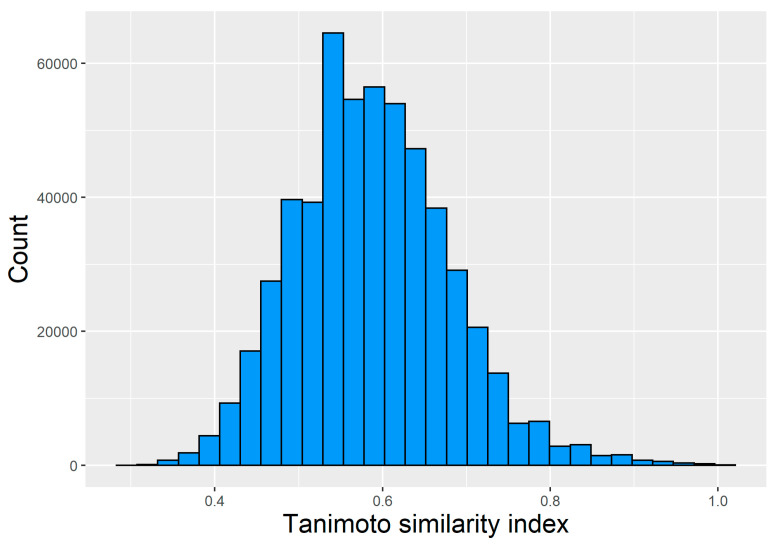
Chemical diversity representation of the HMGCoA-inhibitors dataset—histogram of the Tanimoto similarity (based on MACCS fingerprints) for all possible unique pairs of chemical compounds from the data set.

For the entire data set of HMG-CoA reductase inhibitors (all pairs), the average of the Tanimoto similarity coefficients was 0.59, and the first and third quartiles were 0.52 and 0.59 (Figure 2). For the compounds forming the training, the average of the Tanimoto coefficients was also 0.53, whereas for the testing set, it was slightly higher, 0.59. The Tanimoto coefficients for the whole dataset and the training and test subsets indicate a reasonably large chemical diversity for the compounds used in the modeling.

Table 1 displays the number of failures of Lipinski’s rule of five for both active and inactive compounds (defined as previously mentioned). One-third of the active compounds had no failure, while 19.56% had one failure, 22.46% two failures, and 24.64% three failures. No active compound had four or five failures of Lipinski’s rule. Interestingly, most inactive compounds (71.79%) had no Lipinsky failures.

### 2.2. Regression Models and Their Performance

**Figure 3:**
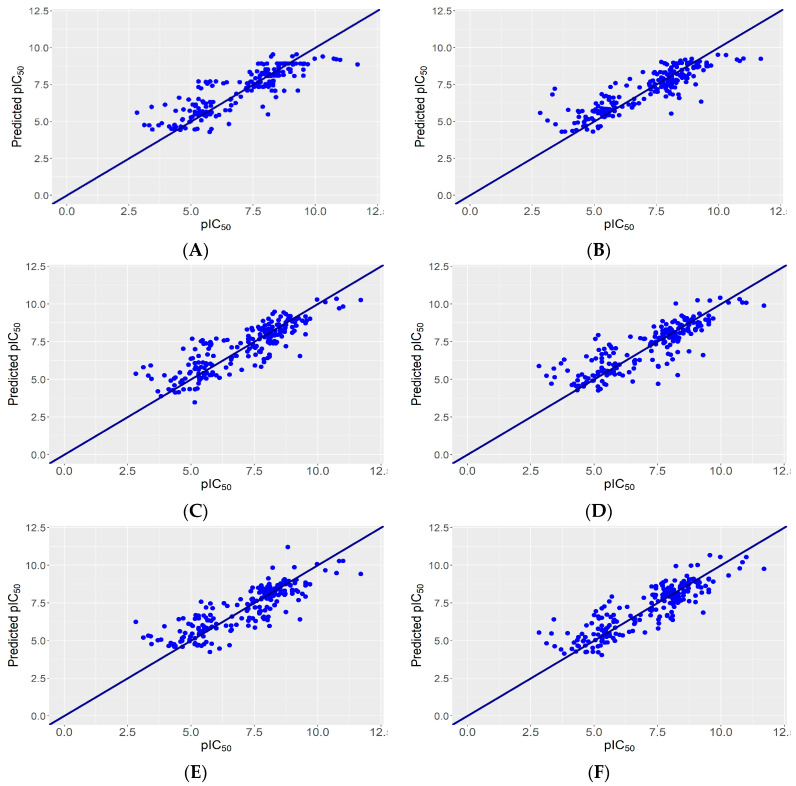
Experimental vs. predicted pIC_50_ for the six best-performing regression models (which were selected to build the ensemble models). The sloping line represents perfect agreement between actual and predicted values. Points above this line indicate overpredictions while points below indicate underpredictions. (**A**): model nr. 4; (**B**): model no. 12; (**C**): model no. 14; (**D**): model no. 15; (**E**): model no. 16; (**F**): model no. 20.

We built and evaluated 300 models through nested-cross validation (using different machine learning regression algorithms, feature selection methods, and fingerprint or descriptor data sets) (Tables S1–S6). From these, we selected 21 models that performed reasonably well in the nested cross-validation (either R^2^ ≥ 0.70 or CCC ≥ 0.85, Table 2 and Figure 3). Among the latter, only six met performance conditions (R^2^ ≥ 0.70 and CCC ≥ 0.85), and these were selected to build five ensemble models. To do this, we used the predicted values from the external loop of the nested cross-validation results (using, as the random seed, the one that gave CCC values closest to the mean value of the five seeds tested; for example, for model no. 4, we used the predicted values for the seed that gave a CCC value of 0.853, as this was the closest to the mean value of 0.851 for that model).

The 21 models with reasonably good performance were based on 7 different algorithms: random forests (five models), BART (five models), boosting algorithms (Xgboost—four models and GBM—one model), support vector machines (three models), rule- and instance-based regression (two models), and weighted k-nearest neighbor (one model). However, the six best performing models were built with the following algorithms: support vector machines (two models), BART (three models), and weighted k-nearest neighbor (one model). With respect to feature selection algorithms, among the six best performing models, three were built with the help of Boruta, two with “gaselect”, and one with the “jmim” algorithm. Among the 21 selected models, 9 were built with Boruta, 7 with “gaselect”, 4 with “jmim”, and 1 with “cmim”. 

**Figure 4:**
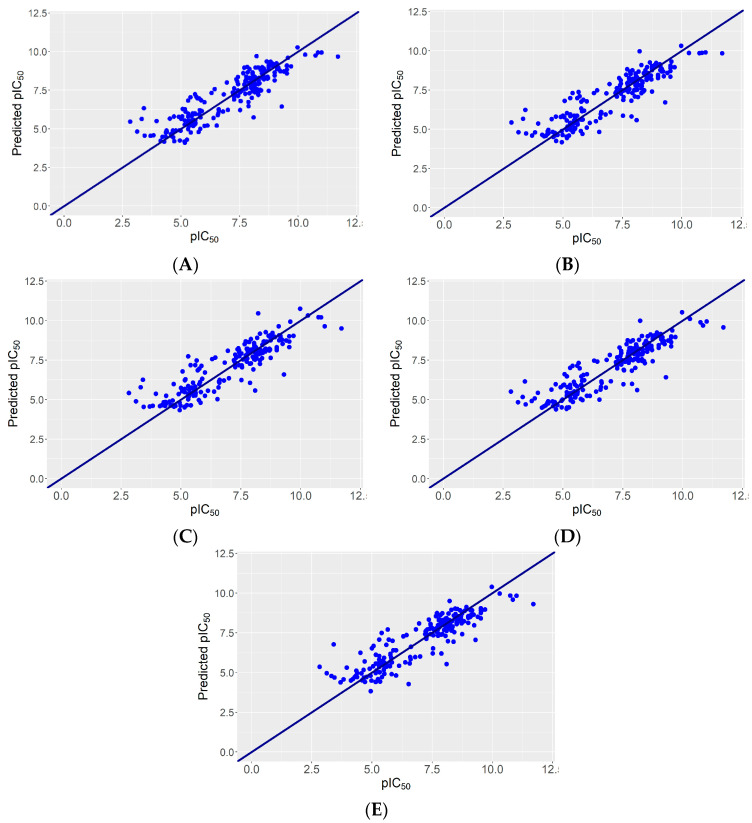
Experimental vs. predicted pIC_50_ for the five ensemble models. (**A**) SVM. (**B**) BART. (**C**) KKNN. (**D**) Random forests. (**E**) Xgboost. The sloping line represents perfect agreement between actual and predicted values. Points above this line indicate overpredictions while points below indicate underpredictions.

We built five ensemble models, each using the predicted values in the external loop of the six best performing models (models no. 4, 12, 14–16, and 20 in Table 2) and five different tree-based algorithms: support vector machines, BART, weighted k-nearest neighbor, random forests, and XGboost. The performance of these ensemble models is shown synthetically in Table 3 and visually in Figure 4.

#### 2.2.1. y-Randomization

To assess the significance of our findings, we conducted a permutation test (y-randomization test). We permuted the response variable in the initial dataset and thereafter followed the same procedure of feature selection and nested cross-validation as for the authentic dataset and computed the same performance metrics (Table 4). While in the feature selection with simple cross-validation, a few of the models had reasonable performance, in the nested cross-validation, all three metrics indicated overwhelming underperformance. This provides evidence that the observed performance in the original data is not due to chance but rather reflects genuine relationships between the features and the response variable.

In the literature, it has been proposed that an $R_{p}^{2}$ value should be computed as $R_{p}^{2}=R^{2}\times \sqrt{R^{2}-R_{r}^{2}}$, where R^2^ is the value of the non-random model and the $R_{r}^{2}$ is the mean R^2^ value of the randomized models. An ideal QSAR model should have $R_{r}^{2}$ close to zero and and $R_{p}^{2}$ close to R^2^ for the genuine model [32]. The y-randomization tests and the $R_{p}^{2}$ values have convincingly confirmed that the models selected are not the result of mere chance.

#### 2.2.2. Descriptors Useful for HMGCo-A Inhibition Prediction

We used the DALEX and iml R packages to look at the most important factors that explained the HMGCoA inhibition in the six best models.

The most important MACCS keys identified in the best-performing model used with these types of descriptors (model no. 4 in Table 2), along with their structural significance and impact on activity, are shown in Table 5 and Figures S1 and S2.

For the model based on the second set of descriptors (2D matrix-based descriptors, 2D autocorrelations, and Burden eigenvalues) (model no. 12 in Table 2), the descriptors most strongly associated with the HMGcoA reductase inhibition are summarized in Table 6 and Figures S3 and S4. For each key descriptor in the model, we have also provided the descriptors that are highly correlated with it, as a key descriptor may simply be a proxy for other highly correlated descriptors or, alternatively, may be more intuitively understood in this way, thus facilitating the interpretation of its contribution. The activity relationship is described on the basis of the partial dependence plots; although such plots are useful for understanding the way in which a feature is associated with the response variable, they may not capture the full complexity of the interactions between the contributing features [33].

For model no. 14, built with descriptors from set 4 (functional group counts, atom-centred fragments, atom-type E-state indices, and pharmacophore descriptors) and using BART as a regression algorithm and “jmim” as a feature selection method, the most important descriptors are summarized in Table 7 and Figures S5 and S6.

For model no. 15, built with descriptors from set 4 (functional group counts, atom-centred fragments, atom-type E-state indices, and pharmacophore descriptors) and using KKNN as the regression algorithm and “Boruta” as the feature selection method, the most important descriptors are summarized in Table S7 and Figures S7 and S8.

For model no. 16 (Table 2), built with descriptors from set 4 (functional group counts, atom-centred fragments, atom-type E-state indices, and pharmacophore descriptors) and using BART as the regression algorithm and “gaselect” as the feature selection method, the most important descriptors are summarized in Table S8 and Figures S9 and S10.

For model no. 20, built with descriptors from set 4 (functional group counts, atom-centred fragments, atom-type E-state indices, and pharmacophore descriptors) and using BART as the regression algorithm and “Boruta” as the feature selection method, the most important descriptors are summarized in Table S9 and Figures S11 and S12.

#### 2.2.3. Virtual Screening of a Data Set of Natural Compounds

**Figure 5:**
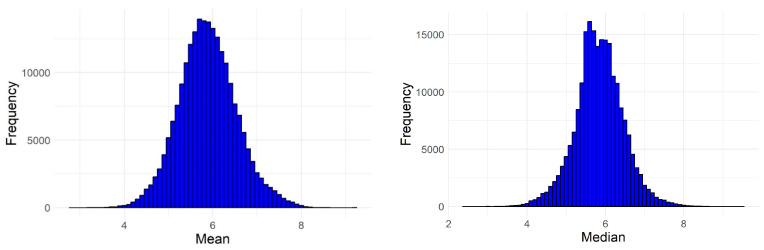
Histograms illustrating the distribution of the predicted mean (**left**) and median (**right**) pIC_50_ values for the 219,897 screened natural compounds from the ZINC 15 database.

We have used the six best-performing models (and the best ensemble model (svm)) to virtually screen a set of nearly 220,000 chemical compounds (mostly natural) from the ZINC 15 database [34]. The distribution of the mean predicted pIC_50_ values is shown in Figure 5. Only 237 compounds had a mean of reliable pIC_50_ predictions (i.e., inside the AD) equal to or greater than 8, and 287 had a median of reliable predictions greater than 8 (i.e., had IC_50_ values equal to or lower than 10 nM). Using the svm-based ensemble model, 168 compounds (about 0.08%) had predicted IC_50_ values lower than 10 nM.

**Figure 6:**
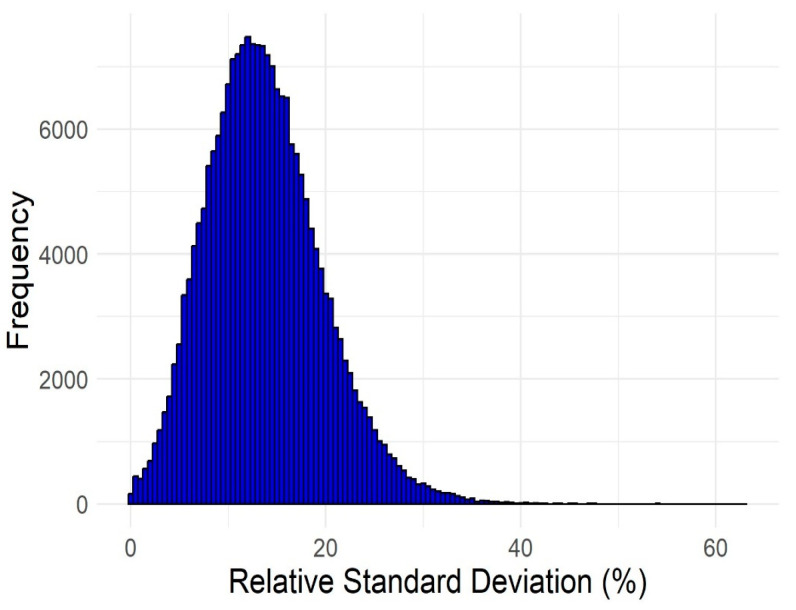
Distribution of the relative standard deviations (RSD) of the predictions made by the six selected models for each of the 219,897 virtually screened compounds.

The distribution of the relative standard deviation (RSD, which expresses how well the predictions for each compound agree with each other) for the virtually screened compounds is shown in Figure 6. The mean and median RSD were approximately 13% (13.27% and 13.74%), the minimum RSD was 0.04%, and the maximum RSD was 63.16%. For most compounds, the predictions were relatively close to one another; for 75% of the predictions, the RSD was less than 17.5%, i.e., there was at least moderate agreement for about three quarters of the data. However, this also means that in about a quarter of the cases, despite the selection of models with similar performance, the predictions differed to a sizeable extent. 

A total of 81,508 compounds (37.07% of all compounds screened) were inside the AD for all 6 models, and 88,046 compounds (40.04%) were inside the AD for 5 of the 6 models. At the other extreme, 1758 compounds (0.80%) were outside the AD for all 6 models, 4550 (2.07%) were inside the AD for a single model, and 9143 (4.16%) were inside the AD for only 2 models.

#### 2.2.4. Use Case Example for Herbal Extracts

**Figure 7:**
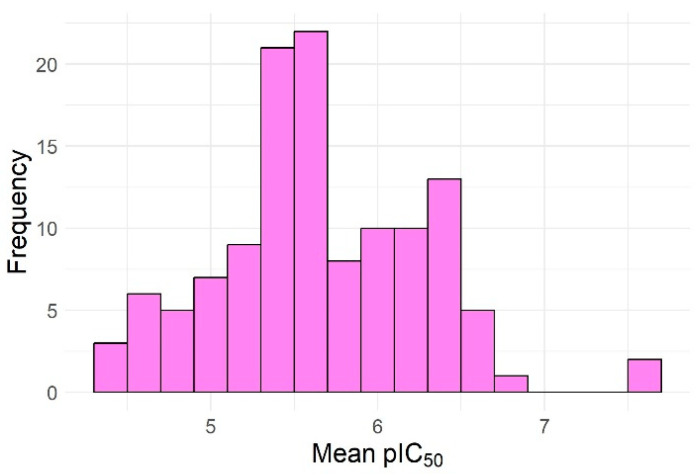
Histogram of the predicted pIC_50_ values for the chemical compounds reported in *Iris × germanica* L.

**Figure 8:**
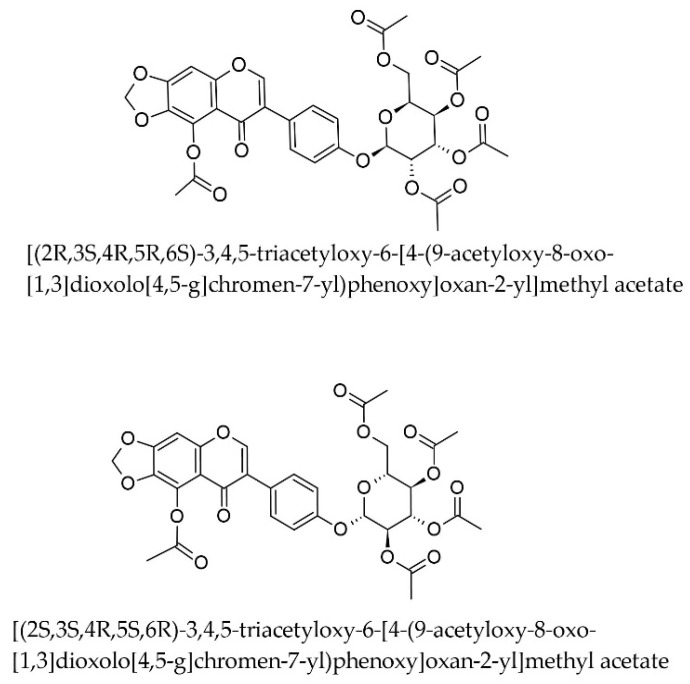
Two acetylated isoflavonoids from the rhizome of *Iris* × *germanica* predicted to be highly active against HMGCoA-reductase.

A study by Iqbal Choudhary et al. (2005) found that an ethanolic extract of Iris germanica L. (rhizomes) significantly lowered all lipid components, including LDL-cholesterol [30]. The authors did not identify or discuss the chemical compounds responsible or the mechanism of action, and we were unable to identify further published research to clarify this aspect. We were therefore interested in assessing whether compounds biosynthesized by Iris × germanica have the ability to inhibit HMGCoA reductase. To this end, we downloaded all the chemical compounds reported to date as having been identified in this species from the Lotus database of natural compounds and obtained a dataset of 129 compounds that were virtually screened using our selected models in a similar manner to those from ZINC. Seven compounds from this dataset were outside the AD of all six models and twelve compounds were inside the AD of a single model among the six. Sixty (46.5%) compounds were inside the AD of all six models, and thirty-eight (29.5%) were inside the AD of five out of the six models. The models predicted only two compounds, both stereoisomers of the same acetylated isoflavone basic structure, to have an IC_50_ less than 100 nM, while no compounds in this dataset had an IC_50_ less than 10 nM (Figure 7). The two were [(2R,3S,4R,5R,6S)-3,4,5-triacetyloxy-6-[4-(9-acetyloxy-8-oxo-[1,3]dioxolo [4,5-g]chromen-7-yl)phenoxy]oxan-2-yl]methyl acetate and [(2S,3S,4R,5S,6R)-3,4,5-triacetyloxy-6-[4-(9-acetyloxy-8-oxo-[1,3]dioxolo[4,5-g]chromen-7-yl)phenoxy]oxan-2-yl]methyl acetate (Figure 8), with a predicted mean IC_50_ of 25.07 nM (RSD 11.57%; the median of the predictions for these compounds was about 36.78 nM, and the predicted IC_50_ made by the svm-based ensemble model was 60.26 nM).

**Figure 9:**
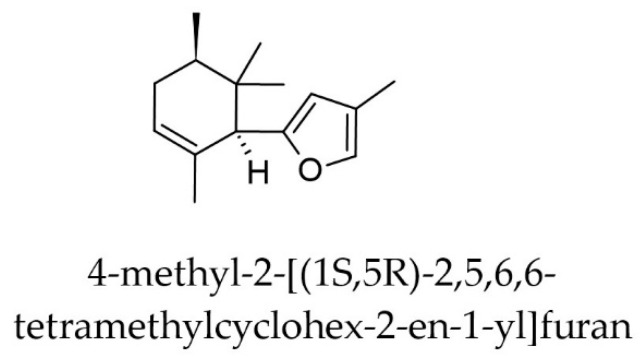
A sesquiterpene derivative from *Iris × germanica* L. with a predicted IC_50_ of 37.2 nM.

The second most active compound of *Iris* × *germanica* predicted by the six models was 4-methyl-2-[(1S,5R)-2,5,6,6-tetramethylcyclohex-2-en-1-yl]furan (Figure 9), a sesquiterpene derivative with a median predicted IC_50_ of 162 nM and a predicted IC_50_ of 595 nM by the svm-based ensemble model. However, the RSD for the four predictions inside AD in this case was relatively large (27.34%).

There were also several additional compounds for which the median of the predicted IC_50_ by the individual models or the predicted IC_50_ by the svm-based ensemble model were less than 1 μM, and these could also contribute to the observed effect. They are listed in Table 8. Most belong to the isoflavonoid group; a few such additional compounds are flavonoids, terpenoids, and xanthonoids.

## 3. Discussion

The QSAR models reported here were built with 2D descriptors and not 3D. Despite the temptation to consider 2D descriptors inferior to 3D ones, previous studies have demonstrated that 2D descriptors could outperform 3D descriptors in compound discrimination across various datasets of biologically active compounds [35]. This was three decades ago, and with the progress made in compound alignment and molecular encoding, this might no longer be true. While 3D-QSAR techniques have multiple strengths, the 2D-QSAR approach still has several advantages: it is simpler, faster, and better suited for analyzing many compounds and screening large molecular databases [36]. Moreover, the performance of 2D-QSAR models can often be very similar to that of 3D-QSAR models [36]. Moreover, the performance of 2D-QSAR models can often be very similar to that of 3D-QSAR models [37]. Therefore, our focus was on developing a set of valid global 2D QSAR models for virtual screening purposes, using two sets of molecular descriptors: the MACCS keys and a variety of molecular descriptors computed by Alvadesc.

Despite their simplicity, the MACCS keys could be used to build a model that performed similarly to models built with more sophisticated descriptors. This is consistent with Brown and Martin’s findings that MACCS keys achieve the highest encoding of content for a variety of properties relevant to interaction with biological targets, such as hydrophobicity, static electricity, steric interactions, dispersion interactions, and intermolecular bonding [35]. In classification models, MACCS (as well as PubChem) fingerprints have been shown to outperform other fingerprints [38]; in our regression models, MACCS yielded results only slightly inferior to the best models constructed with different sets of molecular descriptors.

Among the molecular descriptors, only two pooled sets resulted in models with a reasonably good performance, as defined in this paper: a pooled set consisting of 2D autocorrelations, 2D-matrix based descriptors, and Burden eigenvalues (one model) and another consisting of functional group counts, atom-centered fragments, atom-type E-state indices, and pharmacophore descriptors (four models).

The 2D autocorrelation descriptors characterize the distribution of specific atomic properties throughout the topology of a molecule. The fundamental concept is to measure the correlation of certain atomic attributes (e.g., atomic mass, electronegativity, polarizability etc.) at varying topological distances (i.e., the number of bonds between two atoms in the molecular graph). Because of the autocorrelation function used in their estimation, the topological distance is expressed as a “lag” (e.g., lag 1—atoms directly connected by one bond, lag 2—atoms separated by two bonds, etc.) [39].

Previously, 2D-autocorrelations have been used successfully to build QSAR models for the bioconcentration factor [40], radical scavenging activity [41], muscle relaxant activity [42], matrix metalloproteinase inhibition, and others [43,44]. This family of descriptors is relatively easy to compute and is based on summations of different autocorrelation functions (at different lags) and encodes information about the topology of the molecule or of certain parts of the molecule, as well as certain atomic properties corresponding to that topology [43]. More specifically, the best-performing models included several descriptors calculated from the chi matrix (SM3_X), Laplace matrix (TI2_L), and Burden matrix (SpMax_B(p), VE1sign_B(s)).

MATS3e (Moran autocorrelation of lag 3 weighted by Sanderson electronegativity) has often been identified in the literature as a “potent” descriptor capable of characterizing a variety of ligand-protein interactions [45]. It has been speculated that compounds with higher values of this descriptor have higher electronegative functionalities that favor the formation of hydrogen-bond interactions with amino acid residues of the target protein active site [46]. In the case of our models, more negative values of MATS3e tended to be associated with a more pronounced inhibitory effect. From a structural point of view, more negative values tend to indicate greater differences in electronegativity between atoms separated by three bonds. Its values tended to correlate well with MATS3s (Moran autocorrelation of lag 3 weighted by I-state).

MATS1p, which stands for Moran autocorrelation of lag 1 weighted by polarizability, encodes information about the distribution of polarizability in a molecule, namely between neighboring atoms (lag 1). In the literature, it was reported to correlate positively with the inhibitory activity of imidazole derivatives on glutaminyl cyclase [47] or the inhibitory activity on type I fatty acid synthase [48]. The relationship between MATS1p and the inhibitory activity on HMGCoA reductase in our model was shaped like an upside-down U.

SpMax_B(p) (leading eigenvalue from Burden matrix weighted by polarizability) is a less intuitive descriptor, being a leading eigenvalue derived from the Burden matrix (a mathematical instrument of representing the interactions between molecule atoms) and weighted by polarizability. It can be thought of as reflecting the contributions of all atoms in the molecule, and thus reflecting the diversity or similarity of a dataset or database [49]. Its correlation with the inhibitory effect on HMGCoA reductase has an inverted-U-shape (concave-down).

SpMin1_Bh(e) (smallest eigenvalue n. 1 of Burden matrix weighted by Sanderson electronegativity) belongs to the Burden eigenvalues and seems to have been little used in published QSAR models up to date. One study reported that it is negatively correlated with the binding affinity for the bacterial *LasR* protein [50]. We found that it has a negative association with HMGCo-A inhibitors, with an asymmetric inverted U-shape.

VE1sign_B(s) (coefficient sum of the last eigenvector from Burden matrix weighted by I-State) is a 2D-matrix based descriptor that has rarely been reported as important in QSAR studies. In a recent study, it was found to be the second most important descriptor in describing the activity of aromatase inhibitors [51]. Higher (positive) values of this descriptor were linked to more toxicity in a QSAR study that looked at how harmful chemicals were to the springtail *Folsomia candida* [52]. In our model built with Set 2 of the descriptors, higher values were predictive of lower activity.

SM3_X (spectral moment of order 3 from chi matrix) was not up to date though was reported as an important descriptor in the QSAR literature. It provides information on the structural complexity of the molecule and could reflect certain electronic properties of the molecule. While SM3_X is less intuitive and does not lend itself to easy interpretation, at least in our dataset, it was highly correlated with SM5_X, as well as with the number of 3-membered rings and the distance/detour ring index of order 3/SRW03 (self-returning walk count of order 3), meaning that the presence of 3-membered rings (e.g., epoxides, aziridines, or cyclopropane groups) tends to be associated with lower pIC_50_ (i.e., less active compounds). In a recent study, it was found that nR03 (a descriptor highly correlated with SM3_X) tended to decrease the toxicity of chemical compounds on *Daphnia magna* [53]. In the regression model constructed using the Set 2 descriptors (2D matrix-based descriptors, 2D autocorrelations, and Burden eigenvalues) (model no.12 in Table 1), it showed a negative correlation with pIC_50_.

GATS5v (Geary autocorrelation of lag 5 weighted by van der Waals volume) is a 2D autocorrelation descriptor that encodes information about molecular size, shape, steric effects, and distribution of the van der Waals volume across the molecule, specifically for atoms separated by five bonds (lag 5). It has been shown to be an important predictor for the antagonistic activity of non-peptide compounds against the CXCR2 chemokine receptor [54] as well as for the antiproliferative activity of 3,4-dihydropyrimidin-2-(1H)-thiones [55]. In our model, higher GATS5v values were associated with higher HMGCoA reductase inhibitory properties

JGI5, or mean topological charge index of order 5, is a type of topological index whose values tend to rise as the molecular structure becomes more complicated, with more branching, more ring systems, and more heteroatoms. In the literature, JGI5 has been shown to have a positive association with antimalarial activity [56] or a stronger antioxidant activity [57]. In our model, higher values of JGI5 were associated with a higher inhibitory activity on HMGCoA reductase.

TI2_L (second Mohar index from Laplace matrix) is calculated as the inverse of the smallest non-null eigenvalue of the Laplace matrix, weighted by the amount of heavy (non-hydrogen) atoms. It ignores the presence of heteroatoms in a molecule, but it is sensitive to structural properties such as branching and the presence of rings. Its value increases with the amount of non-hydrogen atoms present. In a set of molecules of the same size, it discriminates between linear chains (higher values) and branched/cyclic structures (lower values). It has been shown to be useful in predicting the biodegradability of molecules [58] and the permeability of the placental barrier [59]. Higher values of TI2_L are associated with a lower inhibitory activity, suggesting that some degree of branching or cyclicity is required for the HMGCoA reductase inhibition.

Atom-centered fragments (ACF) belong to the general group of substructure descriptors, encoding the local chemical environment surrounding an atom within a molecule. These descriptors consider the central atom and its neighboring atoms up to a specified topological distance, providing a detailed representation of the molecule’s structural features [39].

Among the atom-centered fragments, C-034 (R–CR..X, where X is a non-carbon heavy atom, while R is an aliphatic group) and C-003 (a CHR3 group) were shown to correlate with the inhibitory activity on HMGCoA. C-034 correlated well with several other descriptors (see Table 6), including the number of pyrrole rings, which was itself selected as a useful descriptor in other models. Higher values of C-034 were associated with increased activity. C-034 has been reported in the literature to be useful in predicting the glutaminyl cyclase inhibitory activity for imidazole derivatives [47]. C-033 (R–CH..X) has a similar effect as C-034. In a previously published model, it was found to be the most important in predicting herbicidal activity [60], but also in predicting radiosensitizing properties [61]. C-003 was found to be relevant for the binding of small molecules to the active site or the pockets of vasoactive metalloproteases [62] and in predicting the inhibitory activity of biphenylsulfonamides on aggrecanase-1 [56]. In our models, a value of 3 or less was associated with lower activity on HMG-CoA reductase, whereas values of 4 or 5 were associated with higher activity on the enzyme.

C-001 (corresponding to the number of methyl groups, which can induce a certain degree of lipophilicity [63]) has been used in published QSAR models for acetylcholinesterase inhibitors [64]. It was found that both C-001 and the number of pyrrole rings (nPyrroles) were weakly linked to the ability to stop HMG-CoA reductase. Published QSAR models do not appear to have previously selected the number of pyrrole rings among their descriptors. C-002, an atom-centered fragment describing the number of CH_2_R_2_ fragments, had a sawtooth-like relationship with the HMG-CoA reductase inhibitory activity, with the strongest activity being observed at the lowest value for these fragments. In published QSAR models, this descriptor was used in modeling linear retention indices for essential oil constituents [65] and the antagonistic activity of chemical compounds against the growth hormone secretagogue receptor [66]. C-006 (CH2RX, i.e., the number of carbon atoms bonded to two hydrogen atoms, a heteroatom, and another carbon atom) is a descriptor that has been used in previous research to model the MMP-13 inhibitory activity [67], the CK2 inhibitory activity [68], or the aqueous solubility of chemical compounds [69]. In our models, a higher value for this descriptor tended to be associated with lower inhibitory activity on HMG-CoA-reductase.

H-046 (defined as H attached to C0(sp3) with no X attached to the next C, i.e., a hydrogen atom joined to a carbon atom that is saturated (sp3 hybridized), with the subsequent carbon atom unattached to a heteroatom), is an atom-centered fragment descriptor that has been used to model ligand binding to the 5-HT_6_ receptor [70], the inhibitory activity against CDK2 [71], or the PPARγ agonistic activity [72]. In our models, a sawtooth-like curve represented the link between this descriptor and the inhibitory activity of HMG-CoA-reductase, with the highest activity observed at the lowest values. H-053 (defined as H attached to C0(sp3) with 2X attached to the next C; in other words, H-C-C(XX), where: C is an sp3 carbon and C(XX) represents the neighboring carbon with two heteroatoms) is another atom-centered fragment that, in previous research, has been used in QSAR modeling of serotonin 1A and adrenaline α1-adrenergic receptor binding activity [73], of human beta-secretase inhibitors [74], and of the antibacterial activity for pleuromutilin derivatives [75]. In our models, a flattened inverted U-shape was observed for this descriptor in relationship to HMG-CoA reductase inhibitory activity. The O-056 descriptor (number of alcohol fragments) was negatively associated with the HMG-CoA reductase inhibitory activity. It has been used in previously published research to model the odor aroma of wine components [76] or the antimicrobial activity of newly synthesized chemical compounds [77]. The number of pyrimidines (nPyrimidines) correlated positively with the HMG-CoA reductase inhibition. In the past, this descriptor has also been shown to correlate with hepatotoxicity [78] and with the CYP2C9-drug interaction [79].

nCrt (number of ring tertiary C) belong to the functional group counts and was previously reported to be a useful predictor of P-glycoprotein substrates [80]. A value of zero for nCrt was associated with higher HMG-CoA reductase inhibitory activity, whereas values of 1 or higher, were associated with lower activity. NsF (number of atoms of type sF, i.e., single bond fluoride) was also relevant for the HMG-CoA reductase inhibition, with fluorinated molecules having a higher activity. This is an aspect that has already been discussed in the literature, where a fluorine substituent in the pyrrole nucleus of atorvastatin is more effective than other ligands, and fluorine substituents in the hydrophilic side-chain of other statins have stronger inhibitory effects on the target enzyme [81].

nCconj (the number of non-aromatic conjugated carbon atoms, C(sp^2^)), is a descriptor that indicates the count of carbon atoms in a molecule that are sp^2^ hybridized (have a planar structure with a double bond), are involved in a conjugated system, and are not part of an aromatic ring. It has been shown to be useful in predicting the larvicidal activity of terpenoids against *Culex quinquefasciatus* [82] or the activity against *Trypanosoma cruzi*, the causative agent of the Chagas disease [83]. In our models, a higher number of non-aromatic conjugated carbon atoms was associated with greater inhibitory activity on HMG-CoA reductase.

SaaaC is an E-state descriptor, more specifically the sum of aaaC E-states, i.e., aromatic carbon atoms that have no hydrogen atoms attached and are bonded to three other aromatic atoms; the higher its value, the higher the reactivity and number of those carbon atoms. SaaaC has been shown to be negatively associated with the inhibitory activity against bacterial biofilms [84]. The same type of relationship was observed in our models (lower values of this descriptor are associated with an increase in activity). Conversely, greater values of SaaCH (the sum of aaCH E-states, i.e., all the non-substituted carbon atoms in an aromatic molecule) were correlated with slightly increased activity. This descriptor has previously been used to model algal toxicity [85] and cytotoxicity on the MCF-7 breast cancer cell line [86]. SssCH2 (sum of ssCH2 E-states, i.e., electrotopological states of a methylene group attached to the remainder of the molecule through single bonds) has been useful in modeling the histone deacetylase inhibition activity [87] and in modeling the critical micelle concentration (CMC) for anionic surfactants [86]. A slightly lower level of activity was associated with higher values of this descriptor in our models.

Pharmacophore descriptors are molecular blueprints that highlight the essential features required for a molecule to interact with a biological target. They identify the key functional groups and their spatial arrangement within a molecule. CATS2D descriptors (Chemically Advanced Template Search) are a type of 2D pharmacophore descriptor that quantifies the topological distances between different pharmacophore features within a molecule. The typical features include hydrogen bond donors (D), hydrogen bond acceptors (A), positively charged groups (P), negatively charged groups (N), and lipophilic groups (L). The descriptors are generally labeled as CATS2D_NN_AB, where “NN” indicates the topological distance (different digits) and “AB” specifies the particular combination of pharmacophoric features [88].

CATS2D_04_AA (CATS2D Acceptor-Acceptor at lag 04) belongs to the sub-block of CATS (Chemically Advanced Template Search) 2D descriptors in the pharmacophore descriptor block. A value of 3 or higher is associated with stronger inhibitory activity on HMG-CoA reductase. In a recent paper, it was shown that CATS2D_04_AA is an important predictor of blood–brain barrier permeability [89], as well as skin permeability [90] for different substances. CATS2D_04_DA (CATS2D Donor-Acceptor at lag 04) belongs to the same descriptor block and (at least in our dataset) was well correlated with CATS2D_04_AA. It has been used in previous studies to construct quantitative structure–toxicity relationship models [91] and in modeling the inhibitory activity of chemical compounds against the MAO-B enzyme [92]. CATS2D_07_DA (CATS2D Donor-Acceptor at lag 07, i.e., at a distance of seven bonds) was used to model the inhibitory activity of O6-methylguanine-DNA methyltransferase, where higher values correlated with lower activity [93]; the same type of relationship was also seen in our models. CATS2D_07_DL (CATS2D Donor-Lipophilic at lag 07) has been used in published QSAR models for *Aedes aegypti* repellents [94], models for antioxidant activity of coumarin derivatives [95], or the anticancer activity of N-(aryl/heteroaryl)-4-(1H-pyrrol-1-yl)-benzenesulfonamide derivatives [96]. In our models, the inhibitory activity against the HMG-CoA reductase was associated with higher values of this descriptor. CATS2D_06_AL (CATS2D Acceptor-Lipophilic at lag 06) is a descriptor that has been little used to date in QSAR models; we have only identified one model where it was used in the chemometric analysis of drug groups with various pharmacological activities [97] and another model where it was used in modeling the antioxidant effects (TEAC) of chemical compounds [98]. Higher values of this descriptor tended to be associated with lower inhibitory activity on HMG-CoA reductase. CATS2D_03_DL (CATS2D Donor-Lipophilic at lag 03) has been used to model toxicity of chemical compounds against bees [99] and the binding affinity of substances with endocrine disruptor properties [100], whereas CATS2D_09_DL (CATS2D Donor-Lipophilic at lag 09) seems to have not been part of QSAR models published to date. An increase in activity was associated, in our models, with lower values of these two descriptors. CATS2D_02_AL (CATS2D acceptor-lipophilic at lag 02, i.e., two bonds apart) is another pharmacophore descriptor that has been used in modeling the biological activities of SGLT2 inhibitors [101] and the multiple endpoint acute toxicity of chemical compounds (higher values, higher toxicities) [102].

First proposed in 2006 [103], Shannon entropy descriptors have not seen extensive use in QSAR models to date. SHED_AN (Shannon entropy descriptor, acceptor-negative) is a descriptor that offers information regarding the spatial arrangement of acceptor and negative atoms inside the molecule. To date, it has been used in models predicting the blood-brain barrier permeability [104]. Higher SHED_AN values in our models were linked to marginally lower activity. Similarly, SHED_AA (Shannon entropy descriptor, acceptor-acceptor) is an expression of the diversity or uniformity of the acceptor-acceptor interactions (acceptors being generally electronegative atoms, e.g., halogens, oxygen, and nitrogen). Lower values of SHED_AA were associated with higher HMG-Co-A inhibitory activity in our research.

How do our models compare with other published models? Rajathei et al. (2020) built their models on only 30 pyrrole derivatives of atorvastatin, and the R^2^ estimated on an external dataset was only 0.64, our results being considerably superior [25]. Moorthy et al. (2015) reported slightly better results than ours (with R^2^ext of 0.83 for the best model versus 0.79 for the best of our ensemble model), but their validation relied on the methodologies of leave-one-out (LOO), leave-many-out (LMO), and bootstrapping, in addition to randomization and holdout testing [26]. Nested cross-validation, used in our papers, is more apt to provide a reliable estimation of model performance and a better control of overfitting. Furthermore, that article used a larger number of compounds, but it does not clarify whether the HMG-CoA reductase inhibitors were assessed on a human or rodent variant of the enzyme. Samizo and Kaneko (2023) constructed QSAR models from a dataset of 833 chemicals from the ChEMBL database; however, they employed a rat-derived HMG-CoA reductase rather than a human-derived one [27]. In addition, the performance of their models was slightly inferior to the performance reported in our paper (r^2^ on the external data set of 0.77). Zang et al. (2017) developed a 3D-QSAR model utilizing a limited sample of 19 chemicals, focusing on lepidopteran HMG-CoA reductase rather than human HMG-CoA reductase [28]. For their ComFA model, a q^2^ of only 0.66 was reported. Finally, a QSAR model built on a small number (*n* = 18) of phthalimide congeners had an r^2^CV of only 0.59 [29]. Compared with other published models, the performance reported here is among the best and is evaluated through a non-biased manner.

As shown in the results, the virtual screening of almost 220,000 chemical compounds (mostly natural) from the ZINC 15 database predicted, for only 237 compounds, a mean of reliable pIC_50_ predictions (i.e., within the AD) equal to or higher than 8, and for 287 compounds, a median of reliable predictions higher than 8 (i.e., had IC_50_ values equal to or lower than 10 nM). Using the svm-based ensemble model, 168 compounds (about 0.08%) had predicted IC_50_ values lower than 10 nM. In a recent paper, Athista et al. (2023) reported using virtual screening to identify HMG-Co-A reductase inhibitors using ligand-protein docking; their predicted hit rate was 22 natural compounds out of 558 compounds tested, i.e., 3.94% [105].

The compounds predicted to be active are of particular interest because they include novel chemical scaffolds that are worth exploring for the synthesis of new compounds. For example, one of the most active predicted compounds is an isomer of yuanhuapin, a daphnane derivative. Additionally, two other compounds were derivatives of pyrrolo[3,4-d]pyrimidine-2,4-dione, which, according to PubChem, have been previously tested for multiple biological effects. Although both were inactive in most bioassays, one of them was found to be active against *Plasmodium falciparum* apicoplast DNA polymerase (Pf-apPOL). Another compound predicted to be highly active features a scaffold consisting of an indole-thiazole-amide system, while yet another has a spiro[piperidine-pyrroloquinoxaline] scaffold. We intend to discuss these predictions in greater detail in a future paper. We have also shown how such QSAR models can be used to improve the understanding of non-clinical experiments performed with herbal extracts where a pharmacological mechanism of the anti-hypercholesterolemiant effect has not been explored. In our use case example, we have identified several natural products from *Iris germanica* L. that could explain the ability of an extract obtained from the rhizomes of this species to reduce LDL-cholesterol. Among the compounds predicted to be active by our models was mangiferin. For this compound, the median of the IC_50_ values predicted by the four best-performing models for which the substance was within the AD was 1.68 μM, whereas experimentally an inhibition constant of 3 ± 0.2 μM was determined [106], which seems to be in fairly good agreement. The ensemble model based on svm estimated an IC_50_ of 0.90 μM, which is also close to the experimental inhibition value. For irisolidone, our models predicted IC_50_ values of 0.53 or 1.24 μM, whereas in one experiment, an IC_50_ of 36 μM was estimated [107]. Such examples, where we have found experimental evidence to verify the predicted activity, tend to confirm the validity of the models and their usefulness in this setting.

## 4. Materials and Methods

### 4.1. Data Set

A set of 1170 of human HMG-CoA reductase inhibitors, whose activity was assessed on the basis of their half-maximal inhibitory concentration (IC_50_), was downloaded from ChEMBL (target ID CHEMBL402) [108]. The SMILE chemical formulae were carefully checked manually, and inorganic or overly simple compounds (e.g., sodium arsenite, strontium chloride hexahydrate, thioacetamide, etc.), polymers (e.g., macrogol), mixtures, or other compounds without a defined chemical structure were removed from the dataset. ChemAxon Standardizer 18.8.0 (ChemAxon, Budapest, Hungary) was used to standardize the chemical structure of the compounds in the dataset using the following operations: stripping salts, neutralization, tautomerization, aromatization, clean 2D, and adding explicit hydrogens (in this order). After standardization, duplicate compounds were removed from the dataset, and their IC_50_ values were replaced by the median (as this is more relevant than the mean in the presence of outliers). Compounds available in both acid and salt forms (e.g., lovastatin and lovastatin sodium, maduramicin and maduramicin ammonium) were treated as duplicates, retaining the acid form. This was conducted using DataWarrior (v. 6.1.0) [109], Flare^TM^ for Academics, v.7.0 (Cresset^®^, Litlington, Cambridgeshire, UK), and the computing and the programming environment R, v. 4.3.1 [110]. After pre-processing operations, the final dataset consisted of 1042 compounds (available with their chemical structures in SMILES notation in Table S1); their IC_50_ values varied between 0.002 nM and 1,500,000 nM, while their molecular weight varied between 32 g mol^−1^ and 2297 g mol^−1^. Of the 1042 compounds, numerical IC_50_ values were available for only 227 compounds, while for the vast majority of the dataset, IC_50_ values were not accessible, and therefore not suitable for use in building regression models. For modelling purposes, the IC_50_ values were converted to pIC_50_ values by taking the negative logarithm (log10) of the corresponding molar concentration. The 227 compounds were randomly divided into training and test datasets in a 3:1 ratio (170 and 57 compounds, respectively).

#### 4.1.1. Molecular Fingerprint Calculation

The R package “Rcpi” (an open source library) [111] was used to compute MACCS keys (166 bits) under Rstudio, v. 2021.09.1, Build 372 [112]. Molecular fingerprints are a mean of representing molecular structures, encoding the presence (assigning a value of 1) or absence (assigning a value of 0) of certain fragments/substructures in a chemical molecule [113]. MACCS fingerprints were originally intended to be used for substructure searching [114], but were later widely used in QSAR modeling and are still relevant for this purpose [115]. AlvaDesc software (v. 1.0.22) [88] was used to compute 3874 2D molecular descriptors, grouped into 18 blocks (constitutional indices, ring descriptors, topological indices, etc.).

#### 4.1.2. Chemical Space Distribution and Diversity

To investigate the diversity and distribution of the dataset compounds in the chemical space, we have used two features widely used in the field: molecular weight and atomic logP (AlogP, AK Ghose-G.M. Crippen logP) [116], computed by the R package “Rcpi” [111]. We also investigated the fulfillment of Lipinski’s “rule of five” as a criterion of “druggability” or “drug-likeness” for the compounds included in the modeling exercise [117], also using the “Rcpi” R package [111]. We used the average Tanimoto similarity index (computed in R with the “proxy” R package [118]) to assess the diversity of the dataset.

#### 4.1.3. Feature Selection, Model Building and Validation

MACCS fingerprints consist of 166 binary features/keys, whereas Alvadesc computes over 4000 1D or 2D descriptors. Both are large numbers that need to be reduced in order to build meaningful models because of the so-called “curse of dimensionality”, which, if not properly addressed, increases the likelihood of modeling noise and obtaining useless models [119]. It is recognized that, in most cases, only a small subset of all descriptors are likely to carry the information essential for developing good mathematical models with a given dataset [22]. Therefore, feature selection is an important step of the QSAR model building process, and an impressive number of methods and algorithms have been developed for this purpose. They are classified as either filter methods (faster and less computationally intensive) or wrapper methods (more robust but more time-consuming and computationally intensive) [120].

For the regression models, we have explored the use of six filter methods through the unified interface “mlr3” [121]: “carscore” (from R package “care” [122]), “correlation”, “cmim” (R package “praznik” [123]), “find_correlation”, “relief” (R package “FSelectorRcpp” [124]), and “information gain” (R package “FSelectorRcpp” [124]). We preceded feature selection by removing constant, quasi-constant (37 features removed), and highly correlated (36 additional features removed) features, using a correlation cut-off of 0.90 and the “FeatureTerminatoR” [125] R package (36 additional features removed). We coupled feature selection with a hyperparameter search and a 10-fold (and in some cases, 5-fold) cross-validation. We used this k-fold cross-validation to enhance the filtering method results, rather than to validate the modeling exercise (we describe and report external validation and nested cross-validation below). We divided the feature filtering methods into three groups: “carscore”, “correlation”, and “cmim” for the first group; “find_correlation”, “relief”, and “information gain” for the second group. We then used the features of the top-performing methods to construct the regression models. To achieve this, we employed the following regression algorithms:

- multiple linear regression
- elastic net regression (“glmnet” R package [126], varying the *alpha* parameter between 0.0001 and 1)
- multivariate adaptive regression splines (“earth” R package [127])
- k nearest neighbors with various kernels (“kknn” [128] and “FNN” R package [129])
- Quinlan M5 rule trees (“Cubist” R package [130] and “RWeka” R package [131]).
- random forests (“ranger” R package [132]), conditional inference trees and conditional random forests (“partykit” [133], “sandwich” [134] and “coin” [135] R packages)
- support vector machines (“e1071” R package [136]) and regularized support vector regression (“LiblineaR” R package [137])
- extreme gradient boosting (“xgboost” R package [138]) and generalized boosting models (“gbm” R package [139])
- Bayesian Additive Regression Trees (BART) (“BART” R package [140]).

For tree-based algorithms (Quinlan M5 rule trees, random forests, extreme gradient boosting, BART), numerical features were used as such (unscaled) in building and assessing the performance of the models. For the remainder of the algorithms used, features were centered and scaled (using the base R function *scale* within the mlr3 pip pipeline).

To estimate the performance of the model-building exercise, we applied a nested-cross validation procedure. Nested cross-validation is a reliable method for assessing machine learning models. It employs two layers of cross-validation: an outer loop and an inner loop. The outer loop divides the data into training and testing sets. Within each training set, the inner loop further divides the data into multiple training and validation sets. Models are trained on the inner training sets and evaluated on the inner validation sets. The model with the best performance from the inner loop is then selected and tested on the outer testing set. This process is repeated for each fold of the outer loop. Nested cross-validation helps to avoid overfitting and offers a more accurate estimate of the model’s ability to generalize compared to standard cross-validation. This approach is particularly useful with limited data (relatively small data sets, such as a few hundred as observations) [141]. We have used an inner loop of 10 folds and an outer loop of 10 folds and tuning the hyperparameters for each model inside the inner loop. We have used the root mean squared error (RMSE) as a scoring function for tuning and the nested cross-validation R^2^ (true q^2^ [142]) as a more easily interpretable performance measure, as well as the concordance correlation coefficient (CCC, computed with the “agRee” R package [143]). We also applied the models built on the external validation dataset using the R2 and the CCC between the true values and those predicted by the models. The CCC was initially proposed by Lawrence I-Kuei Lin in 1989 as a measure of reproducibility [144], but was more recently recommended in the field of QSAR as a more conservative metric, having the property of being “a true external validation measure” (using no information from the training data set) [145,146]. We rejected models for which the R^2^ values for the test set were lower than 0.70; therefore, for those models we did not perform a nested cross-validation. To control for the possibility of good performance due to chance associated with a certain seed number, we have repeated the nested cross-validation five times for each model, with different random seeds.

To estimate the risk of random correlation, a y-scrambling test (described in the literature as “probably the most powerful validation procedure”) [147] was performed on three of the selected models: the model with the highest R^2^ value in the nested cross-validation (R^2^ = 0.75), one among the models with the lowest acceptable R^2^ values (R^2^ = 0.70), and one with an intermediate level for R^2^ value (0.72) (all three models were built with the set 4 of Alvadesc descriptors). For each model, the response variable was permuted 20 times, and the whole model building process was repeated from step zero (scaling, feature selection with the relevant methods, nested cross-validation).

To assess feature importance, identify the most important variables associated with the HMGCoA reductase inhibition in the best models, and interpret those models, the “DALEX” [148] and “iml” [149] R packages were used.

Trustworthy QSAR model applications rely on the *applicability domain* (AD), which is defined in large part by the characterization of the interpolation space [150]. We used the apd_similarity() function from the “applicable” R package [151] to estimate the AD for models built using MACCS fingerprints, which are binary variables; we considered compounds with a similarity larger than 20% versus the training set inside AD. For the molecular descriptors (computed with the Alvadesc software), the Isolation Forest algorithm was used, as implemented in the “isotree” R package [152], with a number of features randomly selected for splitting (“ntry”) of 10. (The same algorithm is borrowed from the “isotree” by the “applicable” R package.)

To perform a virtual high-throughput screening for potential inhibitors of HMGCoA reductase, a library of approximately 220,000 chemical compounds was obtained from the ZINC database. They were downloaded in the SMILES format and the same molecular descriptors used for the training compounds were computed using Alvadesc. We then used the six best performing models to predict the pIC_50_ values for screening chemical compounds. We assessed whether or not each compound fell within the AD of each model and calculated the median and mean of the predictions that could be trusted based on the AD assessment, as well as the relative standard deviation. The latter allows us to understand how much the predictions have varied between the models whose results were selected for pooling (the molecules being within the AD of those models). To illustrate a practical application of the models, we have also downloaded the chemical structures of all chemical compounds reported as identified in the *Iris germanica* L. species in the Lotus database [153], calculated the molecular descriptors, and then virtually screened each compound in a similar way using the ZINC compound dataset.

## 5. Conclusions

We have developed a set of QSAR models for human HMG-CoA reductase inhibitors, employing nested cross-validation as the primary validation method, and utilizing the top-performing models for the virtual screening of approximately 220,000 chemical compounds from the ZINC 15 database. Active substances (IC_50_ < 100 nM) exhibited molecular weights from 369.4 to 778.1 g mol^−1^ and ALogP values ranging from 1.4 to 8.4. In contrast, the ten statins displayed molecular weights between 390.5 and 558.6 g mol^−1^ and ALogP values from 2.1 to 5.5. Three-hundred models were built using various machine learning regression algorithms, feature selection methods, and fingerprints or descriptor datasets. Twenty-one models were selected for their good performance (R^2^ ≥ 0.70 or CCC ≥ 0.85), among which six met both performance criteria and were used to construct five ensemble models. Employing y-randomization and feature selection with basic cross-validation yielded satisfactory performance for some models, nested cross-validation revealed significant underperformance across all performance measures, thus confirming the validity of the selected models. Using the DALEX and iml R packages, the descriptors that were most important in explaining HMGCoA inhibition in the six best-performing models were identified. Only 237 of about 220,000 compounds had a mean pIC_50_ reliable prediction (i.e., within the AD) of 8 or higher, while 287 of the compounds had a median of 8 or higher for reliable predictions (i.e., IC_50_ values equal to or lower than 10 nM). A total of 168 substances (or roughly 0.08%) had predicted IC_50_ values less than 10 nM using the svm-based ensemble model. The developed QSAR models can be successfully applied to understand the compounds involved in cholesterol-lowering activities of herbal extracts, for instance, an extract of *I. germanica* rhizome.

## Figures and Tables

**Table 1:** Number of failures of the Lipinski’s rule of five for the compounds in the dataset.

No. of Failures	Active Compounds	Inactive Compounds
0	46	649
1	27	127
2	31	52
3	34	34
4	0	40
5	0	2

**Table 2:** Results of nested-cross validation for models with reasonably good performance. Five replicates were used with different random seeds.

No.	Regression Algorithm	Descriptor Set	Feature Selection Method	CCC (Nested CV, *n* = 5) Mean (s.d.)	R^2^ (Nested CV, *n* = 5) Mean (s.d.)	RMSE (*n* = 5) Mean (s.d.)
1	Random forest (“ranger”)	MACCS	“cmim”	0.837	0.707	0.872
				0.840	0.716	0.867
				0.846	0.745	0.835
				0.833	0.698	0.884
				0.825	0.720	0.877
				**0.836 (0.008)**	**0.717 (0.018)**	**0.867 (0.019)**
2	XGboost	MACCS	Boruta	0.848	0.726	0.848
				0.861	0.744	0.821
				0.840	0.725	0.873
				0.850	0.701	0.870
				0.848	0.741	0.835
				**0.849 (0.008)**	**0.727 (0.017)**	**0.849 (0.022)**
3	Random forest (“ranger”)	MACCS	Boruta	0.835	0.712	0.890
				0.827	0.696	0.903
				0.833	0.722	0.877
				0.826	0.679	0.916
				0.834	0.707	0.886
				**0.831 (0.004)**	**0.702 (0.016)**	**0.891 (0.018)**
4	Support vector machines	MACCS	Boruta	0.857	0.743	0.832
				0.853	0.740	0.831
				0.857	0.754	0.815
				0.843	0.708	0.872
				0.845	0.738	0.839
				**0.851 (0.007)**	**0.737 (0.017)**	**0.838 (0.021)**
5	Gradient boosting machine (“GBM”)	Set2	Boruta	0.858	0.752	0.815
				0.820	0.681	0.942
				0.827	0.702	0.912
				0.830	0.696	0.915
				0.829	0.667	0.926
				**0.833 (0.015)**	**0.700 (0.032)**	**0.902 (0.050)**
6	Support vector machines	Set2	“jmim”	0.840	0.734	0.854
				0.841	0.727	0.850
				0.850	0.756	0.824
				0.839	0.728	0.849
				0.841	0.746	0.838
				**0.842 (0.004)**	**0.738 (0.012)**	**0.843 (0.012)**
7	BART	Set2	Gaselect	0.846	0.730	0.858
				0.848	0.739	0.833
				0.854	0.745	0.830
				0.850	0.733	0.847
				0.845	0.689	0.864
				**0.849 (0.004)**	**0.727 (0.022)**	**0.846 (0.015)**
8	Random forest (“ranger”)	Set2	Gaselect	0.827	0.733	0.848
				0.830	0.730	0.849
				0.823	0.708	0.864
				0.830	0.742	0.850
				0.818	0.723	0.874
				**0.826 (0.005)**	**0.727 (0.013)**	**0.857 (0.011)**
9	XGboost	Set2	“jmim”	0.832	0.724	0.868
				0.843	0.724	0.852
				0.832	0.706	0.885
				0.830	0.684	0.890
				0.823	0.705	0.901
				**0.832 (0.007)**	**0.709 (0.017)**	**0.879 (0.019)**
10	BART	Set2	Boruta	0.867	0.764	0.797
				0.825	0.690	0.929
				0.825	0.697	0.917
				0.834	0.707	0.901
				0.829	0.674	0.919
				**0.836 (0.018)**	**0.704 (0.034)**	**0.893 (0.054)**
11	Rule- and instance-cased regression	Set2	Gaselect	0.837	0.724	0.860
				0.821	0.707	0.891
				0.835	0.717	0.881
				0.843	0.727	0.851
				0.823	0.660	0.893
				**0.832 (0.009)**	**0.707 (0.027)**	**0.875 (0.019)**
12	Support vector machines	Set2	Gaselect	0.849	0.748	0.821
				0.853	0.754	0.804
				0.840	0.715	0.846
				0.856	0.766	0.804
				0.851	0.757	0.819
				**0.850 (0.006)**	**0.748 (0.020)**	**0.819 (0.017)**
13	Random forest (“ranger”)	Set3	Gaselect	0.812	0.702	0.873
				0.832	0.720	0.841
				0.826	0.727	0.860
				0.826	0.731	0.862
				0.823	0.717	0.876
				**0.824 (0.007)**	**0.719 (0.011)**	**0.862 (0.014)**
14	BART	Set4	“jmim”	0.864	0.751	0.821
				0.845	0.710	0.888
				0.858	0.742	0.844
				0.853	0.731	0.858
				0.852	0.730	0.856
				**0.854 (0.007)**	**0.733 (0.015)**	**0.853 (0.024)**
15	Weighted k-Nearest Neighbor	Set4	Boruta	0.826	0.690	0.923
				0.854	0.740	0.846
				0.865	0.739	0.821
				0.848	0.709	0.874
				0.858	0.737	0.858
				**0.850 (0.015)**	**0.723 (0.022)**	**0.864 (0.038)**
16	BART	Set4	Gaselect	0.856	0.743	0.833
				0.847	0.726	0.865
				0.845	0.715	0.871
				0.846	0.719	0.877
				0.859	0.745	0.848
				**0.851 (0.006)**	**0.730 (0.014)**	**0.859 (0.018)**
17	XGboost	Set4	“jmim”	0.835	0.701	0.857
				0.832	0.702	0.896
				0.856	0.750	0.825
				0.830	0.705	0.902
				0.846	0.737	0.844
				**0.840 (0.011)**	**0.719 (0.022)**	**0.865 (0.033)**
18	Random forest (“ranger”)	Set4	Boruta	0.831	0.702	0.861
				0.846	0.748	0.835
				0.857	0.754	0.796
				0.855	0.749	0.829
				0.847	0.738	0.848
				**0.847 (0.010)**	**0.738 (0.021)**	**0.834 (0.024)**
19	Rule- and instance-cased regression	Set4	Gaselect	0.851	0.726	0.859
				0.813	0.655	0.947
				0.841	0.721	0.882
				0.842	0.715	0.879
				0.837	0.688	0.896
				**0.837 (0.014)**	**0.701 (0.030)**	**0.893 (0.033)**
20	BART	Set4	Boruta	0.856	0.743	0.833
				0.870	0.757	0.814
				0.868	0.743	0.836
				0.872	0.760	0.805
				0.877	0.768	0.800
				**0.869 (0.008)**	**0.754 (0.011)**	**0.818 (0.016)**
21	XGboost	Set4	Boruta	0.850	0.737	0.848
				0.849	0.747	0.834
				0.850	0.734	0.838
				0.855	0.738	0.842
				0.843	0.711	0.893
				**0.849 (0.004)**	**0.733 (0.013)**	**0.851 (0.024)**

**Table 3:** Performance of the five ensemble models.

Ensemble Algorithm	CCC (Nested CV)	R^2^ (Nested Cross-Validation)	RMSE (Nested Cross-Validation)
Support vector machines	0.893	0.798	0.730
BART	0.888	0.789	0.745
KKNN	0.887	0.789	0.750
Random forests	0.889	0.794	0.739
Xgboost	0.883	0.784	0.760

**Table 4:** Performance metrics for three representative data sets and algorithms for which the response variable was permuted 20 times.

Model Whose Features Were Randomized	CCC (Nested CV, *n* = 20) Mean (s.d.)	$R_{r}^{2}$ (Nested CV, *n* = 20) Mean (s.d.)	RMSE (*n* = 20) Mean (s.d.)	$R_{p}^{2}$ (for the Corresponding Model)
Model 19 in Table 2	0.047 (0.055)	−0.220 (0.077)	1.804 (0.045)	0.803
Model 17 in Table 2	−0.007 (0.034)	−0.113 (0.068)	1.731 (0.027)	0.773
Model 20 in Table 2	0.078 (0.060)	−0.056 (0.040)	1.685 (0.032)	0.781

**Table 5:** Important MACCS keys identified in model no. 4 (from Table 2), their corresponding structural patterns, and their association with the HMGCoA inhibitory activity.

MACCS Key	Structural Pattern	Association
62	“A$A!A$A” (any atom—ring bond—any atom—chain bond—any atom—ring bond—any atom)	Positive
85	CN(C)C (a closed ring formed by a C-N-C chain)	Positive
105	“A$A($A)$A” (aromatic atom—substructure—aromatic atom)	Negative
22	Three-membered ring system (3M ring)	Relatively strongly negative
65	Carbon and nitrogen united by an aromatic query bond	Positive
145	6M RING > 1 (more than one six-member rings)	Positive
89	OAAAO (two oxygen atoms connected by three other atoms)	Positive
97	NAAAO (a nitrogen atom connected by a sequence of four single bonds to an oxygen atom)	Weakly negative
107	XA(A)A (where X is a halogen and A any atom)	Weakly positive
42	F (a fluorine atom)	Weakly positive

**Table 6:** Key Descriptors Utilized in the Regression Model Constructed using the Set 2 descriptors (2D matrix-based descriptors, 2D autocorrelations, and Burden eigenvalues) (model no. 12 in Table 2). The model employed SVM as the regression algorithm with the genetic algorithm (“gaselect”) as a feature selection method.

Descriptor	Correlation Coefficient (for Other Descriptors)	Correlated Descriptors	Activity Relationship
MATS3e (Moran autocorrelation of lag 3 weighted by Sanderson electronegativity)	r = 0.846	MATS3s (Moran autocorrelation of lag 3 weighted by I-state)	Negative values → higher activity
SpMax_B(p) (Leading eigenvalue from Burden matrix weighted by polarizability)	r > 0.91 r > 0.80	SpDiam_B(p) (Diameter from Burden matrix weighted by polarizability) SpMax1_Bh(p) (Leading eigenvalue n. 1 of Burden matrix weighted by polarizability) piPC06 (molecular multiple path count of order 6) SpDiam_B(v) (spectral diameter from Burden matrix weighted by van der Waals volume) SpMax_B.v.	Inverted U-shape
VE1sign_B(s) (Coefficient sum of the last eigenvector from Burden matrix weighted by I-State)	N/A	None	Higher values → lower activity
SpMin1_Bh(e) (Smallest eigenvalue n. 1 of Burden matrix weighted by Sanderson electronegativity)	r = 0.99 r > 0.87 −0.80	SpMin1_Bh(i) (Smallest eigenvalue n. 1 of Burden matrix weighted by ionization potential) SpMin1_Bh(v) (Smallest eigenvalue n. 1 of Burden matrix weighted by van der Waals volume) SpMin1_Bh(p) (Smallest eigenvalue n. 1 of Burden matrix weighted by polarizability) WiA_D/Dt (average Wiener-like index from distance/detour matrix)	Negative association with an asymmetric inverted U-shape
SM3_X (Spectral moment of order 3 from chi matrix)	r > 0.90 r = 0.81	nR03 (Number of 3-membered rings) D/Dtr03 (Distance/detour ring index of order 3) SRW03 (Self-returning walk count of order 3) SM5_X (Spectral moment of order 5 from chi matrix) B04[N-S] (Presence/absence of N–S at topological distance 4) B06[O-S] (Presence/absence of O–S at topological distance 6) F06[O-S] (Frequency of O–S at topological distance 6)	Negative correlation with pIC_50_
GATS5v (Geary autocorrelation of lag 5 weighted by van der Waals volume)	r = −0.903 r = 0.80	MATS5p (Moran autocorrelation of lag 5 weighted by polarizability) GATS5p (Geary autocorrelation of lag 5 weighted by polarizability)	Increasing values → higher activity
MATS1p (Moran autocorrelation of lag 1 weighted by polarizability)	r = 0.93 r = 0.87	MATS1v (Moran autocorrelation of lag 1 weighted by van der Waals volume), MATS1i (Moran autocorrelation of lag 1 weighted by ionization potential)	Inverted U-shaped relationship with activity
JGI5 (Mean topological charge index of order 5)	NA	None	Higher values → higher inhibitory activity
TI2_L (Second Mohar index from Laplace matrix)	r > 0.8 for all but none > 0.9	MSD (Mean square distance index (Balaban)) AECC (Average eccentricity) DECC (Eccentric) ICR (Radial centric information index) MaxTD (Max topological distance) S3K (3-path Kier alpha-modified shape index) IDE (Mean information content on the distance equality) HVcpx (Graph vertex complexity index) WiA_Dz(Z) (Average Wiener-like index from Barysz matrix weighted by atomic number) SpPosA_Dz(Z) (Normalized spectral positive sum from Barysz matrix weighted by atomic number) SpMaxA_Dz(Z) (Normalized leading eigenvalue from Barysz matrix weighted by atomic number) SpMAD_Dz(Z) (Spectral mean absolute deviation from Barysz matrix weighted by atomic number) WiA_Dz(m) (Average Wiener-like index from Barysz matrix weighted by mass) SpPosA_Dz(m) (Normalized spectral positive sum from Barysz matrix weighted by mass) SpMaxA_Dz(m) (Normalized leading eigenvalue from Barysz matrix weighted by mass) SpMAD_Dz(m) (Spectral mean absolute deviation from Barysz matrix weighted by mass) WiA_Dz(v) (Average Wiener-like index from Barysz matrix weighted by van der Waals volume) SpPosA_Dz(v) (Normalized spectral positive sum from Barysz matrix weighted by van der Waals volume) SpMaxA_Dz(v) (Normalized leading eigenvalue from Barysz matrix weighted by van der Waals volume) SpMAD_Dz(v) (Spectral mean absolute deviation from Barysz matrix weighted by van der Waals volume) WiA_Dz(e) (Average Wiener-like index from Barysz matrix weighted by Sand)	Higher values → lower inhibitory activity

**Table 7:** Key Descriptors Utilized in the Regression Model Constructed using the Set 4 descriptors (functional group counts, atom-centered fragments, atom-type E-state indices, and pharmacophore descriptors) (model no. 14 in Table 2). The model employed BART as the regression algorithm with the ‘jmim’ as a feature selection method.

Descriptor	Correlated Descriptors	Correlation Coefficient (s)	Activity Relationship
C-034 (R–CR..X)	nPyrroles (number of pyrrole rings), N-073 (Ar2NH/Ar3N/Ar2N-Al/R..N..R), SaasN (sum of aasN E-states), NaasN (number of atoms of type aasN)	R = 0.89–0.90	Higher values → higher activity
SHED_AA (Shannon entropy descriptor, acceptor-acceptor)	SHED_DA (Shannon entropy descriptor, acceptor-acceptor)	r = 0.91	Lower values → higher activity
C-003 (a CHR3 group)	nCt (number of total tertiary C), nCrt (number of ring tertiary C)	r = 0.88–0.99	≤3 → lower activity, 4 or 5 → higher activity
nCrt (number of ring tertiary C)	nCt, C-003, SpMin1_Bh(s) (smallest eigenvalue n. 1 of Burden matrix weighted by I-state)	0.80–0.88	0 → higher activity, ≥1 → lower activity
CATS2D_04_AA (CATS2D Acceptor-Acceptor at lag 04)	F04[O-O] (Frequency of O—O at topological distance 4)	r = 0.81	≥3 → Stronger activity
NsF (number of atoms of type sF, i.e., -F)	nF (number of fluorine atoms), nX (number of halogen atoms), P_VSA_e_6 (P_VSA-like on Sanderson electronegativity, bin 6), F-084 (F attached to C1(sp2)), SsF (sum of sF E-states), NsF (number of atoms of type sF), F01[C-F] (frequency of C—F at topological distance 1), F02[C-F] (frequency of C—F at topological distance 2), F03[C-F] (frequency of C—F at topological distance 3), F07[C-F] (frequency of C—F at topological distance), F08[C-F] (frequency of C—F at topological distance 8)	r > 0.9 or r = 1.0	Fluorinated → higher activity
CATS2D_04_DA (CATS2D Donor-Acceptor at lag 04)	CATS2D_04_DD, F04[O-O]	r > 0.80	Higher values → slightly higher inhibition
SHED_AN (Shannon entropy descriptor, acceptor-negative)	SHED_DN, CATS2D_01_DN (CATS2D Donor-Negative at lag 01), CATS2D_00_NN (CATS2D Negative-Negative at lag 00, i.e., number of negative atoms)	r > 0.90	Higher values → slightly lower activities
CATS2D_02_AL (CATS2D acceptor-lipophilic at lag 02)	F04[O-O]	r = 0.84	Higher values → slightly higher inhibition
CATS2D_09_DL (CATS2D Donor-Lipophilic at lag 09)	CATS2D_02_DL, CATS2D_07_DL, CATS2D_08_DL	r > 0.80	Lower values → higher inhibitory activity

**Table 8:** Compounds predicted to have IC_50_ values under 1 μM (but > 100 nM).

No.	Compound	IC_50_ * (μM)	IC_50_ ** (μM)
**Isoflavonoids**			
**1**	irigenin (5,7,3′-trihydroxy-6,4′,5′-trimethoxyisoflavone)	0.56	1.37
**2**	tectoridin (shekanin; 4′,5-dihydro-6-methoxy-7-(o-glucoside)isoflavone)	0.84	0.72
**3**	irisolidone (4′-O-methyltectorigenin)	0.53	1.24
**4**	iristectorin A	0.89	0.82
**5**	iristectorigenin B	0.54	1.12
**6**	homotectoridin	0.87	0.70
**7**	germanaism A	0.52	
**8**	irilone 4′-O-glucoside	0.53	0.73
**9**	germanaism B	0.64	0.80
**10**	germanaism A	0.52	0.95
**11**	Kakkalidone (irisolidone 7-O-beta-D-glucoside and its stereoisomers)	0.59	0.75
**12**	homotectoridin	0.87	
**13**	irisflorentin	1.73	0.80
**14**	pratensein 7-O-glucopyranoside	2.08	0.82
**15**	germanaism G	2.34	0.82
**16**	3-(3-hydroxy-4,5-dimethoxyphenyl)-7-[(2S,3R,4S,5S,6R)-3,4,5-trihydroxy-6-(hydroxymethyl)oxan-2-yl]oxychromen-4-one	1.24	0.69
**17**	5-hydroxy-3-(3-hydroxy-4,5-dimethoxyphenyl)-7-[(2R,3S,4R,5R,6S)-3,4,5-trihydroxy-6-(hydroxymethyl)oxan-2-yl]oxychromen-4-one	1.41	0.78
**18**	germanaism D	2.44	0.85
**flavonoids**			
**19**	isoswertiajaponin	0.83	0.97
**20**	swertisin (flavocommelitin, 6-C-glucopyranosyl-7-O-methylapigenin)	1.24	0.84
**21**	isoswertisin (isoflavocommelitin, 7-O-methylvitexin)	1.07	0.85
**22**	embigenin	1.30	0.66
**terpenoids**			
**23**	iriflorentan (2Z-2-[(2R,3S,4S)-4-hydroxy-3-(hydroxymethyl)-2-(3-hydroxypropyl)-4-methyl-3-[(3E,5E)-4-methyl-6-[(1R,3S)-2,2,3-trimethyl-6-methylidenecyclohexyl]hexa-3,5-dienyl]cyclohexylidene]propanal)	0.62	1.51
**24**	germanical C (2-[4-hydroxy-3-(hydroxymethyl)-2-(3-hydroxypropyl)-4-methyl-3-[4-methyl-6-(2,5,6,6-tetramethylcyclohex-2-en-1-yl)hexa-3,5-dien-1-yl]cyclohexylidene]propanal)	0.76	1.65
**25**	irisgermanical B (2-[4-hydroxy-3-(hydroxymethyl)-2-(3-hydroxypropyl)-4-methyl-3-[4-methyl-6-(2,2,3-trimethyl-6-methylidenecyclohexyl)hexa-3,5-dien-1-yl]cyclohexylidene]propanal)	0.62	1.51
	xanthonoids		
**26**	mangiferin	1.68	0.91
**27**	irisxanthone	1.84	0.98
**28**	isomangiferin	2.49	0.94

* Median IC_50_ value for each compound, calculated using predictions only from models where the compound falls within the AD. ** IC_50_ values predicted by the ensemble support vector machine model.

## Data Availability

Data is contained within the article and Supplementary Materials.

## Supplementary Materials

The following supporting information can be downloaded at: https://www.mdpi.com/article/10.3390/ph17111448/s1, Figures S1–S12: feature importance for models 4, 12, 14, 15, 16, and 20; Tables S1–S6: Performance of different models constructed with different fingerprints or descriptors and different datasets; Tables S7–S9: key descriptors utilized in the regression models no. 15, 16, and 20. CSV file: Preprocessed dataset and maccs fingerprints; Tab-separated text document: Descriptors computed with Alvandesc for the data set compounds; Tab-separated text document: Descriptors computed with Alvadnesc for compounds reported in *Iris germanica* L. The code used to perform the analyses has been made public through Figshare.
