# Supplementary material for: QSAR Regression Models for Predicting HMG-CoA Reductase Inhibition
Source: Pharmaceuticals (Basel). 2024 Oct 30;17(11):1448. doi: 10.3390/ph17111448 (PMC11597356; doi:10.3390/ph17111448)
Supplement: Supplementary file 1 [file pharmaceuticals-17-01448-s001.zip › Figures S1-S12.pdf]

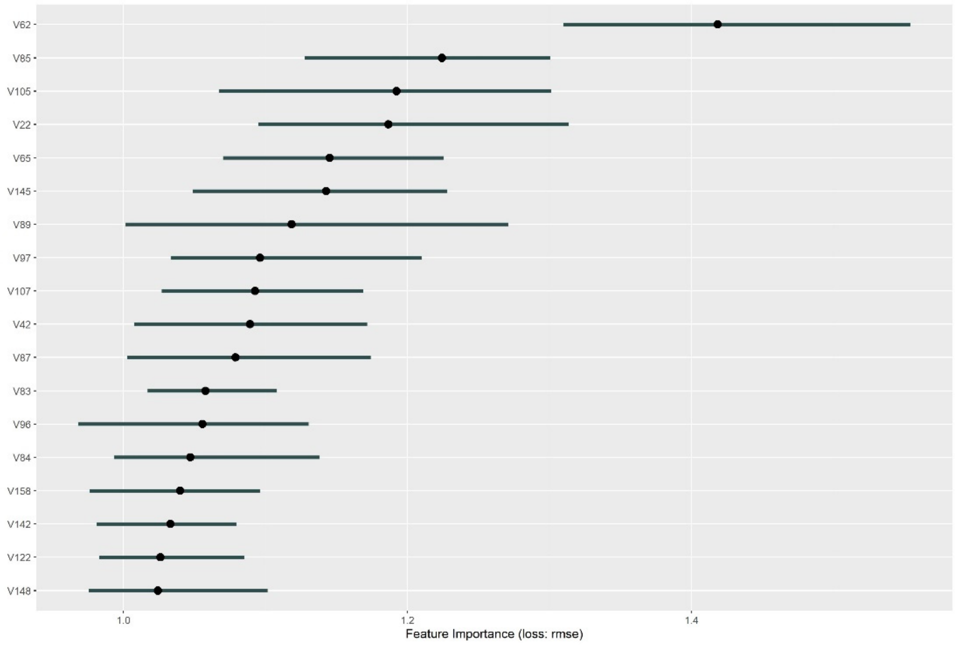

Figure S1. Feature importance for maccs descriptors used in Model 4 (from Table 2). This plot ranks the relative significance of each feature in the model, highlighting which variables exert the most substantial influence on the model's predictions. Features of higher rank contribute more to the model's prediction capability.

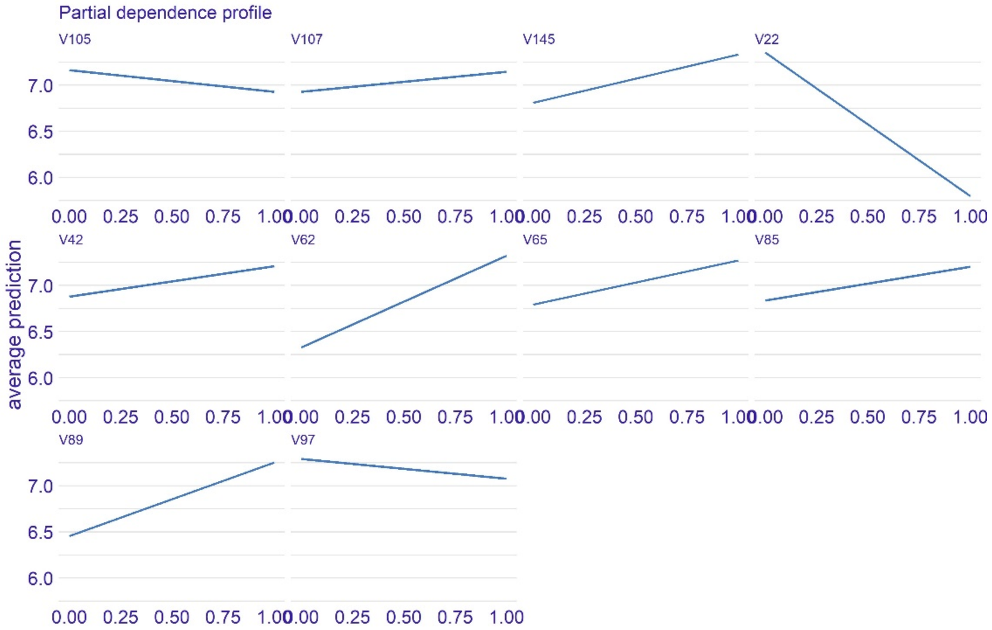

Figure S2. Partial dependence profile plot for the most important features of Model 4 (from Table 2). This plot depicts the correlation between a particular feature and the projected response value, demonstrating how variations in the feature's values affect model predictions while maintaining other variables constant.

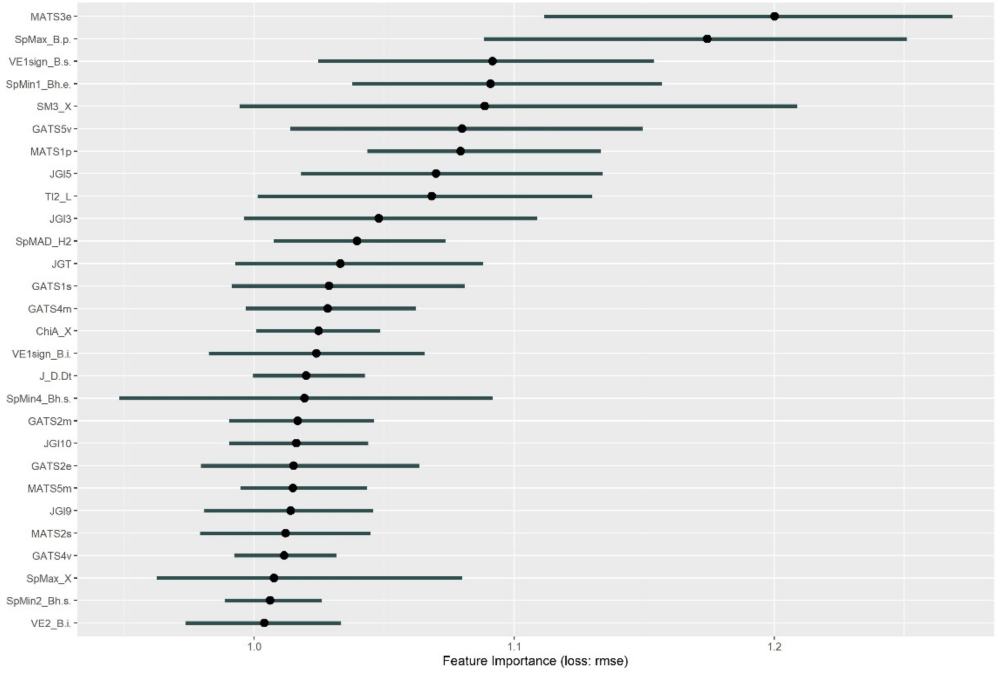

Figure S3. Feature importance for descriptors used in Model 12 (from Table 2). This plot ranks the relative significance of each feature in the model, highlighting which variables exert the most substantial influence on the model's predictions. Features of higher rank contribute more to the model's prediction capability.

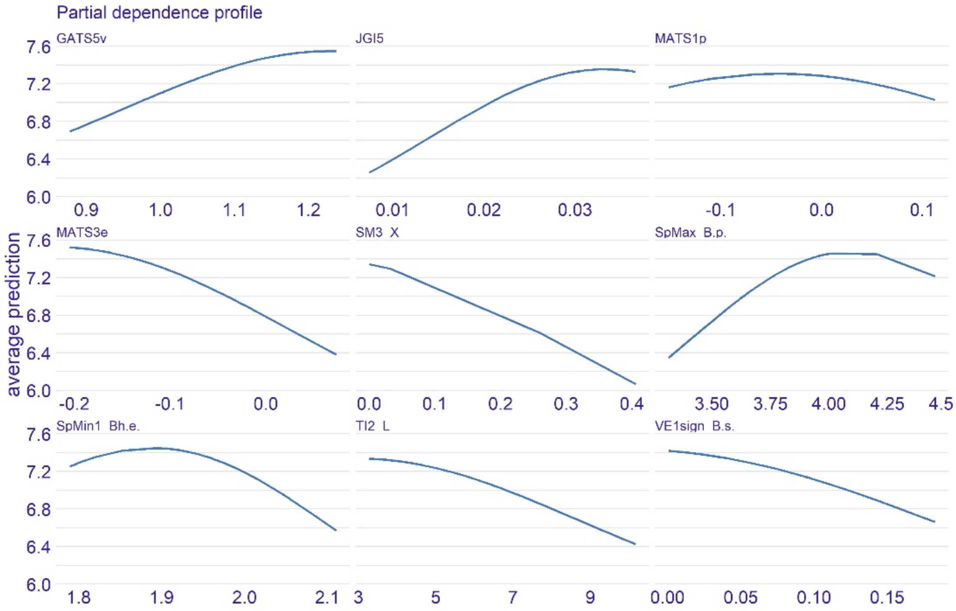

Figure S4. Partial dependence profile plot for the most important features of Model 12 (from Table 2). This plot depicts the correlation between a particular feature and the projected response value, demonstrating how variations in the feature's values affect model predictions while maintaining other variables constant.

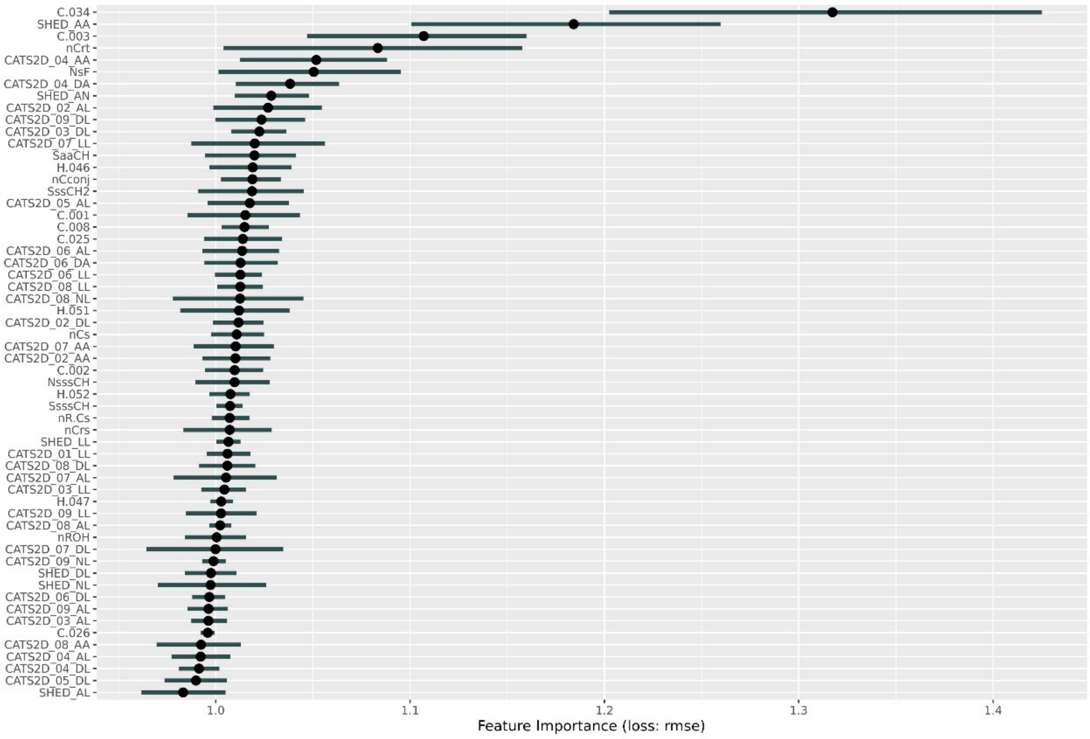

Figure S5. Feature importance for descriptors used in Model 14 (from Table 2). This plot ranks the relative significance of each feature in the model, highlighting which variables exert the most substantial influence on the model's predictions. Features of higher rank contribute more to the model's prediction capability.

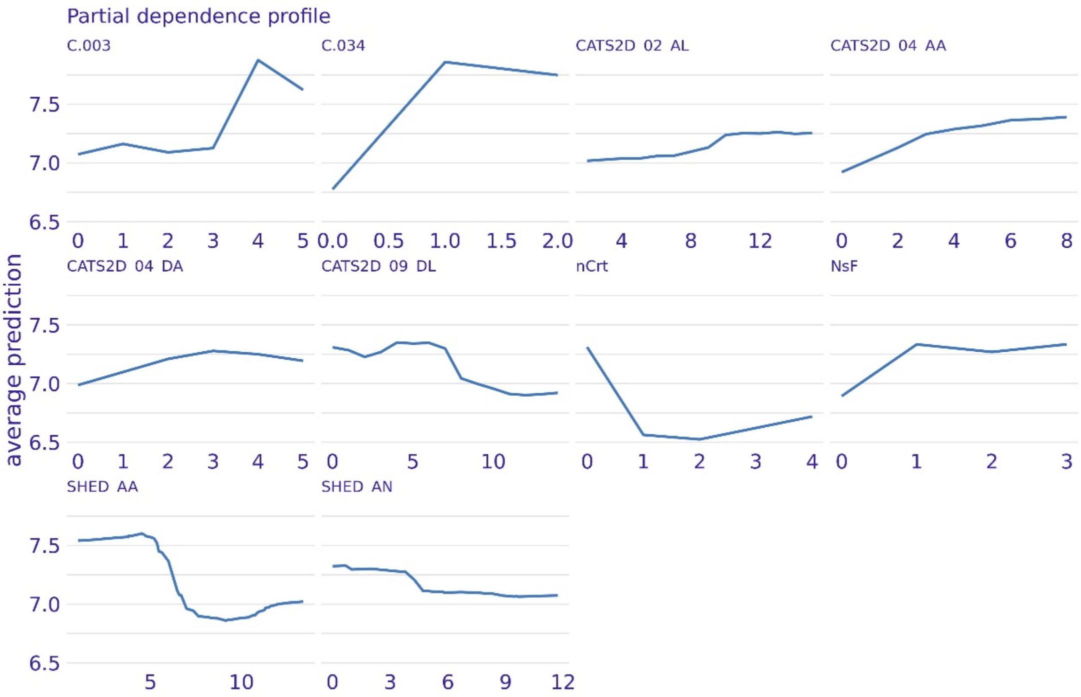

Figure S6. Partial dependence profile plot for the most important features of Model 14 (from Table 2). This plot depicts the correlation between a particular feature and the projected response value, demonstrating how variations in the feature's values affect model predictions while maintaining other variables constant.

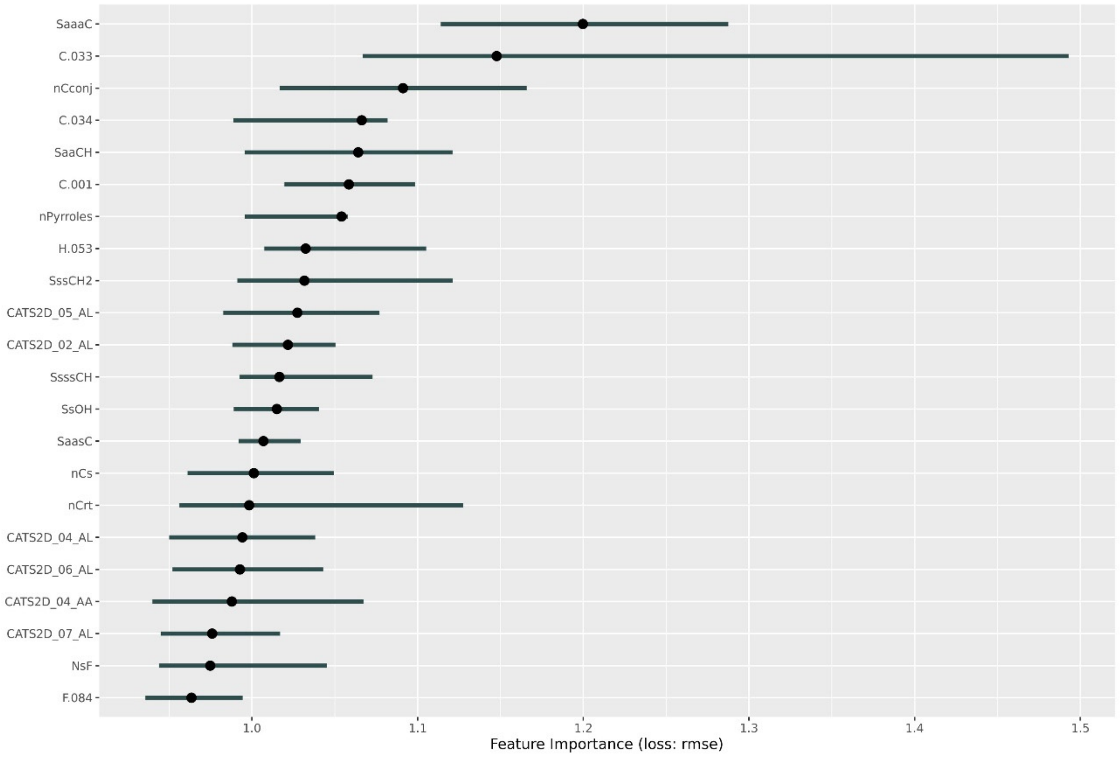

Figure S7. Feature importance for descriptors used in Model 15 (from Table 2). This plot ranks the relative significance of each feature in the model, highlighting which variables exert the most substantial influence on the model's predictions. Features of higher rank contribute more to the model's prediction capability.

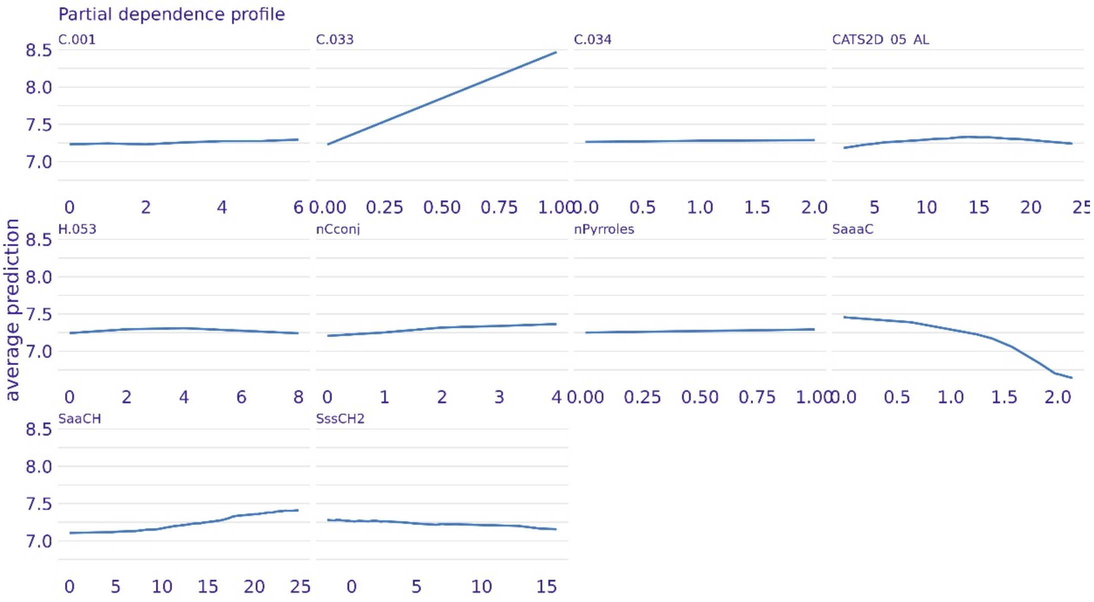

Figure S8. Partial dependence profile plot for the most important features of Model 15 (from Table 2). This plot depicts the correlation between a particular feature and the projected response value, demonstrating how variations in the feature's values affect model predictions while maintaining other variables constant.

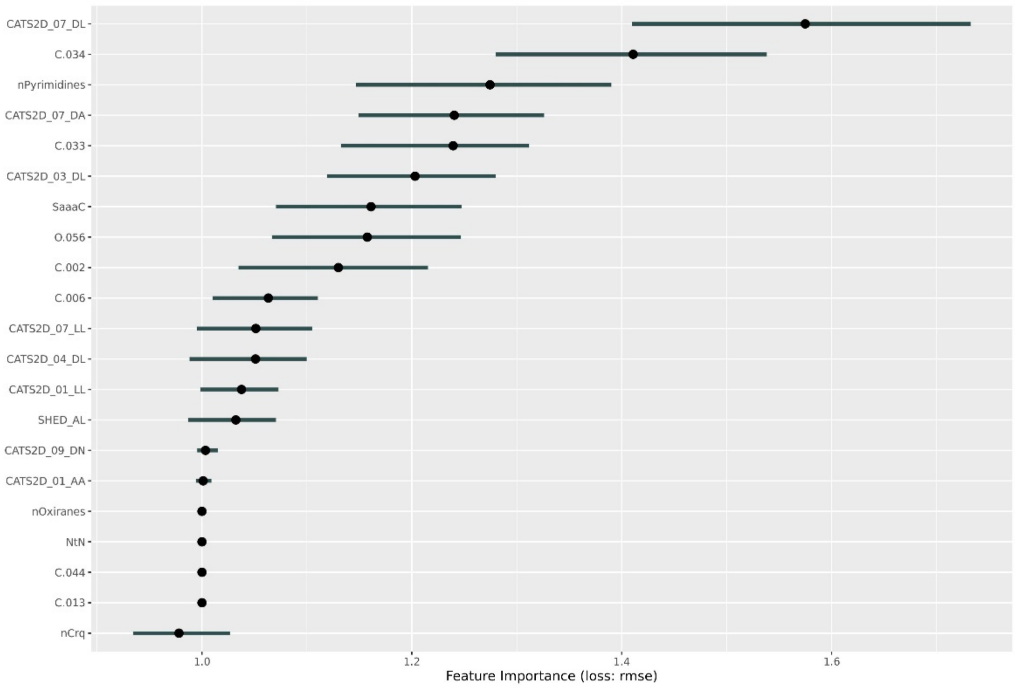

Figure S9. Feature importance for descriptors used in Model 16 (from Table 2). This plot ranks the relative significance of each feature in the model, highlighting which variables exert the most substantial influence on the model's predictions. Features of higher rank contribute more to the model's prediction capability.

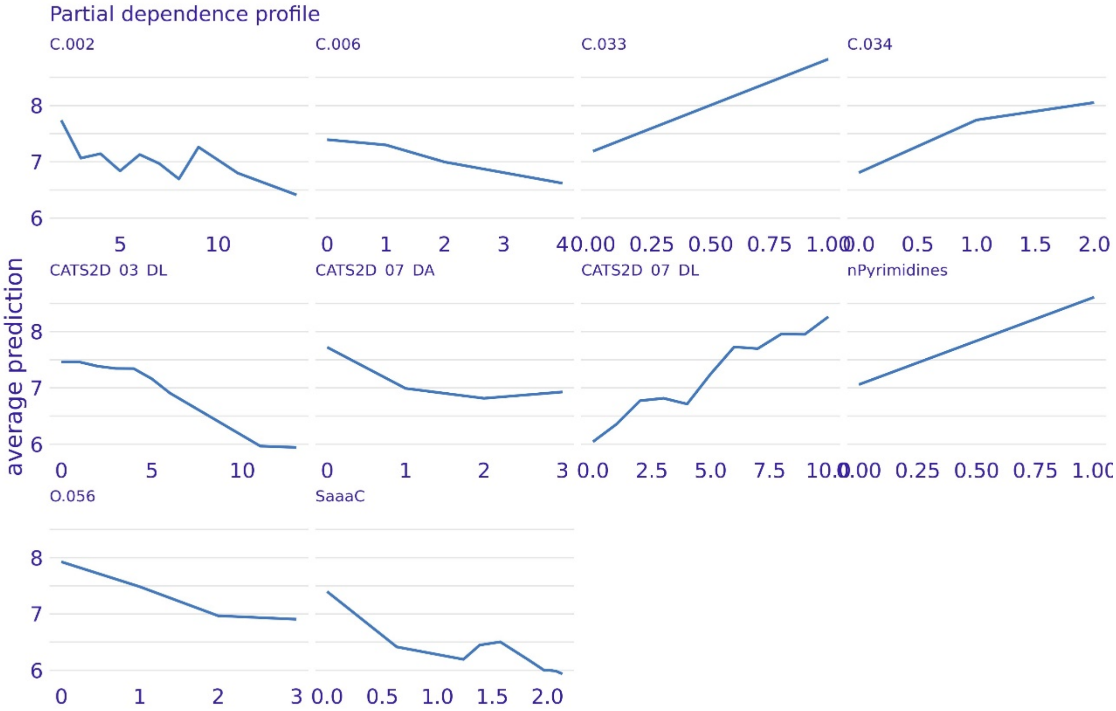

Figure S10. Partial dependence profile plot for the most important features of Model 16 (from Table 2). This plot depicts the correlation between a particular feature and the projected response value, demonstrating how variations in the feature's values affect model predictions while maintaining other variables constant.

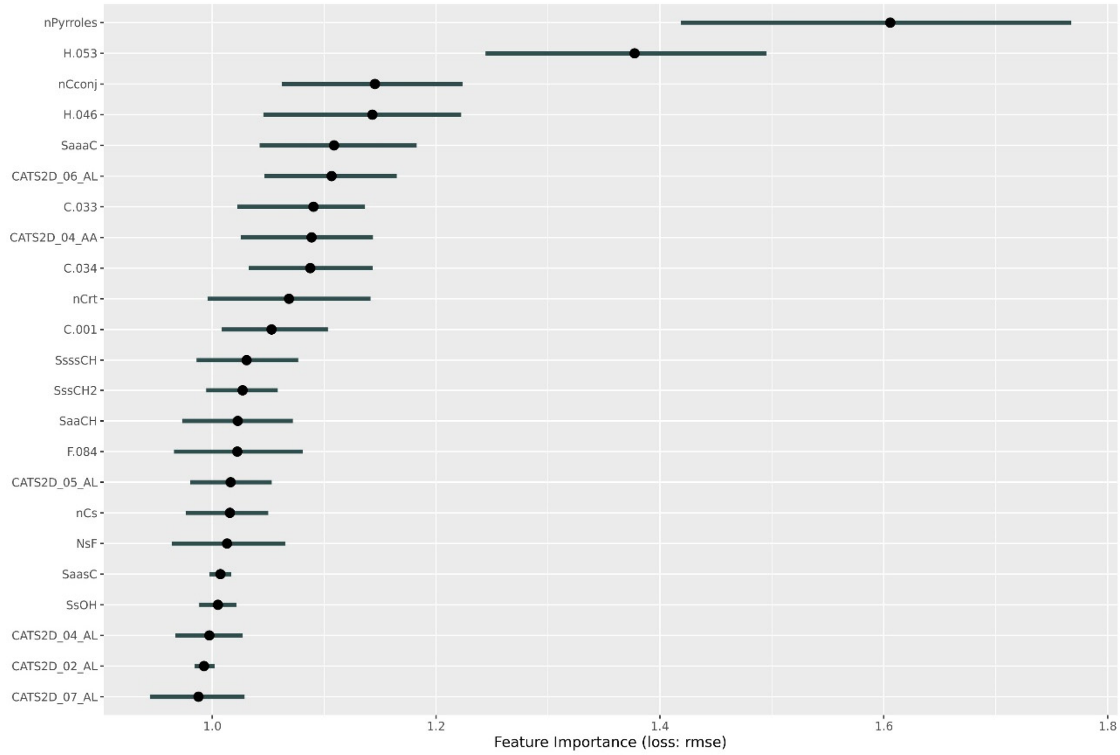

Figure S11. Feature importance for descriptors used in Model 20 (from Table 2). This plot ranks the relative significance of each feature in the model, highlighting which variables exert the most substantial influence on the model's predictions. Features of higher rank contribute more to the model's prediction capability.

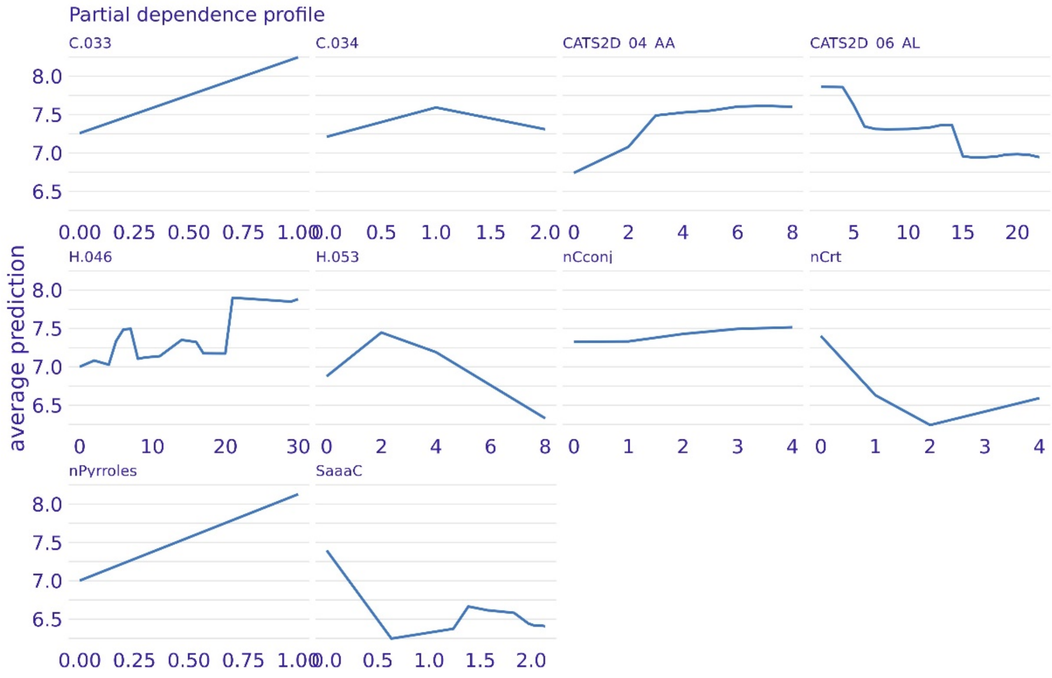

Figure S12. Partial dependence profile plot for the most important features of Model 16 (from Table 2). This plot depicts the correlation between a particular feature and the projected response value, demonstrating how variations in the feature's values affect model predictions while maintaining other variables constant.
