# Supplementary material for: QSAR Regression Models for Predicting HMG-CoA Reductase Inhibition
Source: Pharmaceuticals (Basel). 2024 Oct 30;17(11):1448. doi: 10.3390/ph17111448 (PMC11597356; doi:10.3390/ph17111448)
Supplement: Supplementary file 1 [file pharmaceuticals-17-01448-s001.zip › Tables S1-S6.pdf]

Table S1. Performance of different models built with different regression algorithms and feature selection filters, using the MACCS binary fingerprints

**Regression algorithm**	**Descriptor set**	**Feature selection method**	**R2 (simple CV)**	**R2 (external data set)**	**RMSE (external data set)**	**R2 (nested CV)**	**CCC (nested CV) (95% CI)**	**RMSE (nested CV)**
Linear regression	MACCS	“correlation”	0.673	0.667	0.977	NA	NA	NA
Linear regression	MACCS	“information_gain”	0.736	0.682	0.958	NA	NA	NA
Linear regression	MACCS	“disr”	0.721	0.652	1.003	NA	NA	NA
Linear regression	MACCS	Boruta	0.704	0.630	1.033	NA	NA	NA
Linear regression	MACCS	GASELECT	0.767	0.656	0.996	NA	NA	NA
Glmnet	MACCS	“carscore”	0.566	0.545	1.146	NA	NA	NA
Glmnet	MACCS	“jmi”	0.585	0.573	1.110	NA	NA	NA
Glmnet	MACCS	“disr”	0.669	0.644	1.014	NA	NA	NA
Glmnet	MACCS	Boruta	0.614	0.613	1.057	NA	NA	NA
Glmnet	MACCS	GASELECT	0.656	0.594	1.083	NA	NA	NA
Weighted k-Nearest Neighbor	MACCS	“cmim”	0.832	0.788	0.782	0.602	0.796 (0.726-0.850)	0.980
Weighted k-Nearest Neighbor	MACCS	“find_correlation”	0.908	0.670	0.977	NA	NA	NA
Weighted k-Nearest Neighbor	MACCS	“jmim”	0.862	0.758	0.836	0.650	0.819 (0.751-0.870)	0.920
Weighted k-Nearest Neighbor	MACCS	Boruta	0.861	0.764	0.825	0.662	0.813 (0.737-0.870)	0.964
Weighted k-Nearest Neighbor	MACCS	GASELECT	0.820	0.709	0.917	0.558	0.767 (0.686-0.829)	1.054
Random forest (“ranger”)	MACCS	“cmim”	0.825	0.778	0.801	0.687	0.822 (0.762-0.868)	0.893
Random forest (“ranger”)	MACCS	“jmi”	0.827	0.806	0.748	0.678	0.817 (0.752 –0.867)	0.902

Regression algorithm	Descriptor set	Feature selection method	R2 (simple CV)	R2 (external data set)	RMSE (external data set)	R2 (nested CV)	CCC (nested CV) (95% CI)	RMSE (nested CV)
Random forest ("ranger")	MACCS	"disr"	0.822	0.797	0.766	0.654	0.807 (0.749-0.852)	0.941
Random forest ("ranger")	MACCS	Boruta	0.845	0.798	0.764	0.716	0.849 (0.797–0.888)	0.844
Random forest ("ranger")	MACCS	Gaselect	0.785	0.767	0.820	0.646	0.809 (0.747 - 0.858)	0.936
Support vector machines	MACCS	"correlation"	0.835	0.704	0.924	0.668	0.803 (0.746-0.849)	0.931
Support vector machines	MACCS	"jmi"	0.845	0.783	0.791	0.665	0.806 (0.744–0.854)	0.928
Support vector machines	MACCS	"mim"	0.847	0.760	0.833	0.675	0.816 (0.755-0.864)	0.906
Support vector machines	MACCS	Boruta	0.837	0.754	0.843	0.716	0.844 (0.794-0.883)	0.857
Support vector machines	MACCS	Gaselect	0.753	0.606	1.067	NA	NA	NA
XGboost	MACCS	"cmim"	0.899	0.742	0.864	0.662	0.811 (0.742-0.864)	0.931
XGboost	MACCS	"jmi"	0.813	0.768	0.818	0.655	0.815 (0.753-0.863)	0.931
XGboost	MACCS	"jmim"	0.855	0.735	0.875	0.662	0.812 (0.743-0.865)	0.925
XGboost	MACCS	Boruta	0.858	0.758	0.644	0.705	0.844 (0.784-0.889)	0.875
XGboost	MACCS	Gaselect	0.789	0.685	0.953	NA	NA	NA

Regression algorithm	Descriptor set	Feature selection method	R2 (simple CV)	R2 (external data set)	RMSE (external data set)	R2 (nested CV)	CCC (nested CV) (95% CI)	RMSE (nested CV)
BART	MACCS	“correlation”	0.863	0.784	0.790	0.651	0.815 (0.756-0.861)	0.932
BART	MACCS	“jmi”	0.842	0.796	0.767	0.647	0.810 (0.746-0.858)	0.935
BART	MACCS	“jmim”	0.852	0.786	0.787	0.641	0.814 (0.747-0.865)	0.930
BART	MACCS	Boruta	0.762	0.736	0.872	0.650	0.808 (0.756-0.850)	0.958
BART	MACCS	Gaselect	0.790	0.740	0.781	0.639	0.813 (0.754 - 0.858)	0.950
Multivariate Adaptive Regression Splines (“earth”)	MACCS	“correlation”	0.645	0.569	1.116	NA	NA	NA
Multivariate Adaptive Regression Splines (“earth”)	MACCS	“information_gain”	0.645	0.569	1.116	NA	NA	NA
Multivariate Adaptive Regression Splines (“earth”)	MACCS	“jmim”	0.645	0.569	1.116	NA	NA	NA
Multivariate Adaptive Regression Splines (“earth”)	MACCS	Boruta	0.620	0.602	1.072	NA	NA	NA
Multivariate Adaptive Regression	MACCS	Gaselect	0.633	0.604	1.070	NA	NA	NA

Regression algorithm	Descriptor set	Feature selection method	R2 (simple CV)	R2 (external data set)	RMSE (external data set)	R2 (nested CV)	CCC (nested CV) (95% CI)	RMSE (nested CV)
Splines ("earth")								
Gradient boosting machine ("GBM")	MACCS	"carscore"	0.803	0.758	0.837	0.622	0.796 (0.730-0.848)	0.971
Gradient boosting machine ("GBM")	MACCS	"jmi"	0.768	0.757	0.837	0.625	0.802 (0.729-0.857)	0.951
Gradient boosting machine ("GBM")	MACCS	"mim"	0.827	0.754	0.842	0.658	0.820 (0.757-0.867)	0.915
Gradient boosting machine ("GBM")	MACCS	Boruta	0.786	0.762	0.829	0.668	0.828 (0.774-0.870)	0.902
Gradient boosting machine ("GBM")	MACCS	Gaselect	0.687	0.697	0.935	NA	NA	NA
Regularized suport vector regression ("LiblineaR")	MACCS	"correlation"	0.727	0.671	0.975	NA	NA	NA
Regularized suport vector regression ("LiblineaR")	MACCS	"information_gain"	0.731	0.711	0.913	0.521	0.753 (0.674-0.815)	1.084
Regularized suport vector regression ("LiblineaR")	MACCS	"mim"	0.759	0.703	0.926	0.521	0.755 (0.686-0.810)	1.094
Regularized suport	MACCS	Boruta	0.732	0.680	0.961	NA	NA	NA

**Regression algorithm**	**Descriptor set**	**Feature selection method**	**R2 (simple CV)**	**R2 (external data set)**	**RMSE (external data set)**	**R2 (nested CV)**	**CCC (nested CV) (95% CI)**	**RMSE (nested CV)**
vector regression ("LiblineaR")								
Regularized suport vector regression ("LiblineaR")	MACCS	Gaselect	0.759	0.703	0.927	0.650	0.818 (0.763-0.861)	0.936
Conditional Random Forest	MACCS	"cmim"	0.776	0.758	0.836	0.658	0.820 (0.757-0.867)	0.915
Conditional Random Forest	MACCS	"jmi"	0.737	0.754	0.824	0.640	0.778 (0.723–0.823)	0.968
Conditional Random Forest	MACCS	"mim"	0.765	0.754	0.842	0.622	0.770 (0.709–0.819)	0.988
Conditional Random Forest	MACCS	Boruta	0.776	0.761	0.831	0.670	0.804 (0.757 - 0.843)	0.925
Conditional Random Forest	MACCS	Gaselect	0.695	0.712	0.911	NA	NA	NA
Conditional inference trees	MACCS	"cmim"	0.754	0.707	1.068	0.409	0.697 (0.600 - 0.773)	1.193
Conditional inference trees	MACCS	"jmi"	0.633	0.673	0.972	NA	NA	NA
Conditional inference trees	MACCS	"disr"	0.726	0.610	1.061	NA	NA	NA
Conditional inference trees	MACCS	Boruta	0.719	0.623	1.043	NA	NA	NA
Conditional inference trees	MACCS	Gaselect	0.762	0.751	0.849	0.550	0.754 (0.696 - 0.803)	1.060

Regression algorithm	Descriptor set	Feature selection method	R2 (simple CV)	R2 (external data set)	RMSE (external data set)	R2 (nested CV)	CCC (nested CV) (95% CI)	RMSE (nested CV)
Rule- and instance-cased regression	MACCS	“carscore”	0.798	0.728	0.886	0.590	0.780 (0.711-0.834)	1.032
Rule- and instance-cased regression	MACCS	“information_gain”	0.741	0.736	0.872	0.611	0.777 (0.714-0.827)	1.008
Rule- and instance-cased regression	MACCS	“disr”	0.764	0.755	0.841	0.563	0.747 (0.666 - 0.811)	1.073
Rule- and instance-cased regression	MACCS	Boruta	0.726	0.654	1.000	NA	NA	NA
Rule- and instance-cased regression	MACCS	Gaselect	0.713	0.669	0.978	NA	NA	NA
k-Nearest Neighbor	MACCS	“cmim”	0.802	0.714	0.909	0.561	0.782 (0.703-0.842)	1.022
k-Nearest Neighbor	MACCS	“jmi”	0.825	0.744	0.860	0.554	0.777 (0.699-0.837)	1.075
k-Nearest Neighbor	MACCS	“mim”	0.831	0.745	0.858	0.608	0.799 (0.722-0.856)	0.996
k-Nearest Neighbor	MACCS	Boruta	0.790	0.760	0.832	0.648	0.809 (0.742-0.861)	0.962
k-Nearest Neighbor	MACCS	Gaselect	0.760	0.771	0.813	0.591	0.798 (0.723-0.855)	0.998
M5 rules	MACCS	“correlation”	0.750	0.737	0.872	0.639	0.807 (0.754-0.850)	0.962

Regression algorithm	Descriptor set	Feature selection method	R2 (simple CV)	R2 (external data set)	RMSE (external data set)	R2 (nested CV)	CCC (nested CV) (95% CI)	RMSE (nested CV)
M5 rules	MACCS	“jmi”	0.771	0.702	0.928	0.584	0.783 (0.717-0.835)	1.033
M5 rules	MACCS	“mim”	0.791	0.767	0.821	0.607	0.793 (0.722-0.847)	0.996
M5 rules	MACCS	Boruta	0.769	0.740	0.867	0.672	0.822 (0.766-0.866)	0.921
M5 rules	MACCS	Gaselect	0.759	0.697	0.936	NA	NA	NA

Table S2. Performance of different models built with different regression algorithms and feature selection filters, using different blocks of molecular descriptors computed with Alvadesc

Regression algorithm	Descriptor set	Feature selection method	R2 (simple CV)	R2 (external data set)	RMSE (external data set)	R2 (nested CV)	CCC (nested CV) (95% CI)	RMSE (nested CV)
Linear regression	Set1	“carscore”	0.479	0.403	1.233	NA	NA	NA
Linear regression	Set1	Boruta	0.551	0.496	1.207	NA	NA	NA
Linear regression	Set1	Gaselect	0.689	0.508	1.192	NA	NA	NA
Glmnet	Set1	"find_correlation"	0.380	0.322	1.400	NA	NA	NA
Glmnet	Set1	Boruta	0.506	0.455	1.254	NA	NA	NA
Glmnet	Set1	Gaselect	0.60	0.482	1.223	NA	NA	NA
Weighted k-Nearest Neighbor	Set1	"find_correlation"	0.999	0.664	0.985	NA	NA	NA
Weighted k-Nearest Neighbor	Set1	Boruta	0.999	0.776	0.804	0.673	0.829 (0.760 - 0.880)	0.905
Weighted k-Nearest Neighbor	Set1	Gaselect	0.947	0.598	1.078	NA	NA	NA

Regression algorithm	Descriptor set	Feature selection method	R2 (simple CV)	R2 (external data set)	RMSE (external data set)	R2 (nested CV)	CCC (nested CV) (95% CI)	RMSE (nested CV)
Random forest (“ranger”)	Set1	"find_correlation"	0.926	0.710	0.916	0.656	0.794 (0.730 - 0.845)	0.937
Random forest (“ranger”)	Set1	Boruta	0.939	0.711	0.913	0.651	0.785 (0.720 - 0.835)	0.956
Random forest (“ranger”)	Set1	Gaselect	0.921	0.668	0.980	NA	NA	NA
Support vector machines	Set1	“mim”	0.782	0.622	1.045	NA	NA	NA
Support vector machines	Set1	Boruta	0.756	0.630	1.033	NA	NA	NA
Support vector machines	Set1	Gaselect	0.764	0.554	1.135	NA	NA	NA
XGboost	Set1	“jmim”	0.962	0.742	0.863	0.589	0.781 (0.703 - 0.840)	1.020
XGboost	Set1	Boruta	0.972	0.505	1.196	NA	NA	NA
XGboost	Set1	Gaselect	0.983	0.631	1.032	NA	NA	NA
BART	Set1	“disr”	0.981	0.726	0.889	0.663	0.829 (0.780 - 0.869)	0.915
BART	Set1	Boruta	0.830	0.675	0.969	NA	NA	NA
BART	Set1	Gaselect	0.981	0.672	0.973	NA	NA	NA
Multivariate Adaptive Regression Splines (“earth”)	Set1	“disr”	0.688	0.633	1.030	NA	NA	NA
Multivariate Adaptive Regression Splines (“earth”)	Set1	Boruta	0.688	0.633	1.030	NA	NA	NA

**Regression algorithm**	**Descriptor set**	**Feature selection method**	**R2 (simple CV)**	**R2 (external data set)**	**RMSE (external data set)**	**R2 (nested CV)**	**CCC (nested CV) (95% CI)**	**RMSE (nested CV)**
Multivariate Adaptive Regression Splines (“earth”)	Set1	Gaselect	0.622	0.521	1.176	NA	NA	NA
Gradient boosting machine (“GBM”)	Set1	“disr”	0.979	0.736	0.873	0.658	0.823 (0.765 - 0.868)	0.919
Gradient boosting machine (“GBM”)	Set1	Boruta	0.959	0.667	0.981	NA	NA	NA
Gradient boosting machine (“GBM”)	Set1	Gaselect	0.943	0.638	1.022	NA	NA	NA
Regularized suport vector regression (“LiblineaR”)	Set1	“jmim”	0.720	0.531	1.163	NA	NA	NA
Regularized suport vector regression (“LiblineaR”)	Set1	Boruta	0.571	0.402	1.314	NA	NA	NA
Regularized suport vector regression (“LiblineaR”)	Set1	Gaselect	0.664	0.490	1.214	NA	NA	NA
Conditional Random Forest	Set1	“disr”	0.864	0.717	0.904	0.642	0.776 (0.713 - 0.827)	0.969
Conditional Random Forest	Set1	Boruta	0.877	0.683	0.957	NA	NA	NA

Regression algorithm	Descriptor set	Feature selection method	R2 (simple CV)	R2 (external data set)	RMSE (external data set)	R2 (nested CV)	CCC (nested CV) (95% CI)	RMSE (nested CV)
Conditional Random Forest	Set1	Gaselect	0.596	0.499	1.203	NA	NA	NA
Conditional inference trees	Set1	"jmim"	0.715	0.630	1.034	NA	NA	NA
Conditional inference trees	Set1	Boruta	0.594	0.268	1.454	NA	NA	NA
Conditional inference trees	Set1	Gaselect	0.661	0.394	1.323	NA	NA	NA
Rule- and instance-cased regression	Set1	"jmim"	0.767	0.725	0.892	0.451	0.710 (0.637 - 0.772)	1.178
Rule- and instance-cased regression	Set1	Boruta	0.815	0.717	0.905	0.416	0.681 (0.591 - 0.754)	1.213
Rule- and instance-cased regression	Set1	Gaselect	0.751	0.585	1.095	NA	NA	NA
k-Nearest Neighbor	Set1	"disr"	0.875	0.567	1.118	NA	NA	NA
k-Nearest Neighbor	Set1	Boruta	0.866	0.511	1.189	NA	NA	NA
k-Nearest Neighbor	Set1	Gaselect	0.729	0.411	1.305	NA	NA	NA
M5 rules	Set1	"mim"	0.789	0.728	0.887	0.419	0.659 (0.558-0.740)	1.248
M5 rules	Set1	Boruta	0.761	0.497	1.205	NA	NA	NA
M5 rules	Set1	Gaselect	0.757	0.546	1.145	NA	NA	NA

Table S3. Performance of different models built with different regression algorithms and feature selection filters, using different blocks of molecular descriptors computed with Alvadesc

**Regression algorithm**	**Descriptor set**	**Feature selection method**	**R2 (simple CV)**	**R2 (external data set)**	**RMSE (external data set)**	**R2 (nested CV)**	**CCC (nested CV) (95% CI)**	**RMSE (nested CV)**
Linear regression	Set2	“carscore”	0.802	0.626	1.040	NA	NA	NA
Linear regression	Set2	Boruta	0.601	0.580	1.102	NA	NA	NA
Linear regression	Set2	Gaselect	0.814	0.650	1.005	NA	NA	NA
Glmnet	Set2	"find_correlation"	0.624	0.532	1.163	NA	NA	NA
Glmnet	Set2	Boruta	0.586	0.514	1.185	NA	NA	NA
Glmnet	Set2	Gaselect	0.636	0.548	1.143	NA	NA	NA
Weighted k-Nearest Neighbor	Set2	"jmi"	0.924	0.802	0.757	0.631	0.830 (0.763 - 0.880)	0.95
Weighted k-Nearest Neighbor	Set2	Boruta	1	0.631	1.032	NA	NA	NA
Weighted k-Nearest Neighbor	Set2	Gaselect	0.961	0.762	0.828	0.522	0.788 (0.703 - 0.851)	1.068
Random forest (“ranger”)	Set2	"information_gain"	0.945	0.760	0.833	0.667	0.799 (0.734- 0.850)	0.922
Random forest (“ranger”)	Set2	Boruta	0.964	0.583	1.097	NA	NA	NA
Random forest (“ranger”)	Set2	Gaselect	0.936	0.728	0.886	0.703	0.813 (0.756 –0.859)	0.875
Support vector machines	Set2	“jmim”	0.856	0.760	0.832	0.698	0.825 (0.767 - 0.870)	0.879
Support vector machines	Set2	Boruta	0.839	0.745	0.858	0.638	0.782 (0.713 - 0.837)	0.979
Support vector machines	Set2	Gaselect	0.873	0.744	0.860	0.738	0.858 (0.802 - 0.899)	0.796

**Regression algorithm**	**Descriptor set**	**Feature selection method**	**R2 (simple CV)**	**R2 (external data set)**	**RMSE (external data set)**	**R2 (nested CV)**	**CCC (nested CV) (95% CI)**	**RMSE (nested CV)**
XGboost	Set2	"jmim"	0.993	0.723	0.894	0.709	0.831 (0.775 - 0.874)	0.859
XGboost	Set2	Boruta	0.972	0.763	0.827	0.651	0.787 (0.701 - 0.851)	0.965
XGboost	Set2	Gaselect	0.993	0.726	0.890	0.678	0.836 (0.768 –0.885)	0.866
BART	Set2	"jmim"	0.985	0.703	0.926	0.664	0.823 (0.751 - 0.876)	0.914
BART	Set2	Boruta	0.969	0.701	0.929	0.709	0.857 (0.800 - 0.899)	0.852
BART	Set2	Gaselect	0.965	0.765	0.823	0.700	0.840 (0.778 - 0.886)	0.865
Multivariate Adaptive Regression Splines ("earth")	Set2	"jmim"	0.645	0.333	1.388	NA	NA	NA
Multivariate Adaptive Regression Splines ("earth")	Set2	Boruta	0.638	0.504	1.196	NA	NA	NA
Multivariate Adaptive Regression Splines ("earth")	Set2	Gaselect	0.666	0.374	1.345	NA	NA	NA
Gradient boosting machine ("GBM")	Set2	"mim"	0.831	0.640	1.019	NA	NA	NA

**Regression algorithm**	**Descriptor set**	**Feature selection method**	**R2 (simple CV)**	**R2 (external data set)**	**RMSE (external data set)**	**R2 (nested CV)**	**CCC (nested CV) (95% CI)**	**RMSE (nested CV)**
Gradient boosting machine (“GBM”)	Set2	Boruta	0.992	0.708	0.918	0.694	0.845 (0.786 - 0.888)	0.884
Gradient boosting machine (“GBM”)	Set2	Gaselect	0.963	0.734	0.876	0.667	0.827 (0.765–0.874)	0.915
Regularized suport vector regression (“LiblineaR”)	Set2	“jmim”	0.698	0.588	1.091	NA	NA	NA
Regularized suport vector regression (“LiblineaR”)	Set2	Boruta	0.691	0.608	1.064	NA	NA	NA
Regularized suport vector regression (“LiblineaR”)	Set2	Gaselect	0.815	0.682	0.958	NA	NA	NA
Conditional Random Forest	Set2	“mim”	0.906	0.756	0.840	0.669	0.779 (0.724 - 0.824)	0.935
Conditional Random Forest	Set2	Boruta	0.925	0.747	0.854	0.702	0.817 (0.761 –0.861)	0.890
Conditional Random Forest	Set2	Gaselect	0.755	0.653	1.002	NA	NA	NA
Conditional inference trees	Set2	“mim”	0.760	0.321	1.401	NA	NA	NA
Conditional inference trees	Set2	Boruta	0.800	0.659	0.993	NA	NA	NA

Regression algorithm	Descriptor set	Feature selection method	R2 (simple CV)	R2 (external data set)	RMSE (external data set)	R2 (nested CV)	CCC (nested CV) (95% CI)	RMSE (nested CV)
Conditional inference trees	Set2	Gaselect	0.619	0.589	1.089	NA	NA	NA
Rule- and instance-cased regression	Set2	"jmim"	0.822	0.638	1.022	NA	NA	NA
Rule- and instance-cased regression	Set2	Boruta	0.874	0.648	1.009	NA	NA	NA
Rule- and instance-cased regression	Set2	Gaselect	0.822	0.758	0.835	0.715	0.844 (0.785 - 0.888)	0.852
k-Nearest Neighbor	Set2	"jmim"	0.808	0.747	0.854	0.543	0.788 (0.699 - 0.853)	1.020
k-Nearest Neighbor	Set2	Boruta	1.0	0.642	1.017	NA	NA	NA
k-Nearest Neighbor	Set2	Gaselect	0.836	0.777	0.802	0.513	0.771 (0.678 - 0.839)	1.082
M5 rules	Set2	"mim"	0.752	0.681	0.960	NA	NA	NA
M5 rules	Set2	Boruta	0.663	0.468	1.240	NA	NA	NA
M5 rules	Set2	Gaselect	0.795	0.643	1.015	NA	NA	NA

Table S4. Performance of different models built with different regression algorithms and feature selection filters, using different blocks of molecular descriptors computed with Alvadesc

Regression algorithm	Descriptor set	Feature selection method	R2 (simple CV)	R2 (external data set)	RMSE (external data set)	R2 (nested CV)	CCC (nested CV) (95% CI)	RMSE (nested CV)
Linear regression	Set3	"carscore"	0.694	0.485	1.219	NA	NA	NA
Linear regression	Set3	Boruta	0.518	0.464	1.244	NA	NA	NA

Regression algorithm	Descriptor set	Feature selection method	R2 (simple CV)	R2 (external data set)	RMSE (external data set)	R2 (nested CV)	CCC (nested CV) (95% CI)	RMSE (nested CV)
Linear regression	Set3	Gaselect	0.626	0.566	1.119	NA	NA	NA
Glmnet	Set3	"find_correlation"	0.343	0.428	1.285	NA	NA	NA
Glmnet	Set3	Boruta	0.467	0.528	1.168	NA	NA	NA
Glmnet	Set3	Gaselect	0.570	0.602	1.072	NA	NA	NA
Weighted k-Nearest Neighbor	Set3	“information_gain”	0.881	0.825	0.711	0.634	0.817 (0.750 - 0.868)	0.956
Weighted k-Nearest Neighbor	Set3	Boruta	0.999	0.744	0.859	0.739	0.874 (0.839 - 0.902)	0.785
Weighted k-Nearest Neighbor	Set3	Gaselect	0.896	0.834	0.692	0.666	0.826 (0.757 - 0.876)	0.922
Random forest (“ranger”)	Set3	“information_gain”	0.927	0.809	0.743	0.650	0.796 (0.718 - 0.854)	0.953
Random forest (“ranger”)	Set3	Boruta	0.937	0.775	0.805	0.662	0.801 (0.737 - 0.851)	0.931
Random forest (“ranger”)	Set3	Gaselect	0.941	0.799	0.762	0.702	0.812 (0.753 - 0.858)	0.873
Support vector machines	Set3	“jmim”	0.784	0.723	0.894	0.577	0.740 (0.665 - 0.799)	1.055
Support vector machines	Set3	Boruta	0.782	0.696	0.937	NA	NA	NA
Support vector machines	Set3	Gaselect	0.764	0.702	0.928	0.602	0.749 (0.679 - 0.806)	1.020
XGboost	Set3	“mim”	0.951	0.759	0.835	0.566	0.754 (0.666- 0.821)	1.048
XGboost	Set3	Boruta	0.975	0.771	0.813	0.615	0.800 (0.726 - 0.855)	0.973

Regression algorithm	Descriptor set	Feature selection method	R2 (simple CV)	R2 (external data set)	RMSE (external data set)	R2 (nested CV)	CCC (nested CV) (95% CI)	RMSE (nested CV)
XGboost	Set3	Gaselect	0.964	0.750	0.849	0.626	0.783 (0.704 - 0.843)	0.982
BART	Set3	"disr"	0.970	0.751	0.848	0.638	0.801 (0.728 - 0.856)	0.956
BART	Set3	Boruta	0.884	0.728	0.886	0.580	0.782 (0.704 - 0.841)	1.025
BART	Set3	Gaselect	0.904	0.749	0.852	0.630	0.803 (0.733 - 0.856)	0.951
Multivariate Adaptive Regression Splines ("earth")	Set3	"mim"	0.585	0.551	1.139	NA	NA	NA
Multivariate Adaptive Regression Splines ("earth")	Set3	Boruta	0.618	0.250	1.177	NA	NA	NA
Multivariate Adaptive Regression Splines ("earth")	Set3	Gaselect	0.544	0.609	1.063	NA	NA	NA
Gradient boosting machine ("GBM")	Set3	"jmim"	0.979	0.803	0.753	0.649	0.811 (0.744 - 0.862)	0.946
Gradient boosting machine ("GBM")	Set3	Boruta	0.962	0.788	0.782	0.661	0.826 (0.763 - 0.877)	0.906
Gradient boosting machine ("GBM")	Set3	Gaselect	0.985	0.793	0.773	0.658	0.819 (0.756 - 0.868)	0.934

Regression algorithm	Descriptor set	Feature selection method	R2 (simple CV)	R2 (external data set)	RMSE (external data set)	R2 (nested CV)	CCC (nested CV) (95% CI)	RMSE (nested CV)
Regularized suport vector regression (“LiblineaR”)	Set3	“disr”	0.714	0.564	1.122	NA	NA	NA
Regularized suport vector regression (“LiblineaR”)	Set3	Boruta	0.513	0.471	1. 326	NA	NA	NA
Regularized suport vector regression (“LiblineaR”)	Set3	Gaselect	0.605	0.585	1.094	NA	NA	NA
Conditional Random Forest	Set3	“mim”	0.875	0.754	0.843	0.605	0.751 (0.682 - 0.807)	1.016
Conditional Random Forest	Set3	Boruta	0.669	0.657	1.000	0.719	0.826 (0.772 - 0.869)	0.861
Conditional Random Forest	Set3	Gaselect	0.364	0.368	1.352	NA	NA	NA
Conditional inference trees	Set3	“jmim”	0.656	0.483	1.222	NA	NA	NA
Conditional inference trees	Set3	Boruta	0.766	0.686	0.952	NA	NA	NA
Conditional inference trees	Set3	Gaselect	0.875	0.551	1.138	NA	NA	NA
Rule- and instance-cased regression	Set3	“mim”	0.815	0.695	0.938	NA	NA	NA
Rule- and instance-	Set3	Boruta	0.758	0.687	0.951	NA	NA	NA

Regression algorithm	Descriptor set	Feature selection method	R2 (simple CV)	R2 (external data set)	RMSE (external data set)	R2 (nested CV)	CCC (nested CV) (95% CI)	RMSE (nested CV)
cased regression								
Rule- and instance-cased regression	Set3	Gaselect	0.730	0.709	0.917	0.426	0.680 (0.577 - 0.762)	1.223
k-Nearest Neighbor	Set3	“jmim”	0.700	0.699	0.935	NA	NA	NA
k-Nearest Neighbor	Set3	Boruta	1.000	0.648	1.008	NA	NA	NA
k-Nearest Neighbor	Set3	Gaselect	0.748	0.758	0.835	0.567	0.773 (0.685 - 0.838)	1.062
M5 rules	Set3	“mim”	0.808	0.683	0.957	NA	NA	NA
M5 rules	Set3	Boruta	0.776	0.506	1.194	NA	NA	NA
M5 rules	Set3	Gaselect	0.796	0.528	1.168	NA	NA	NA

Table S5. Performance of different models built with different regression algorithms and feature selection filters, using different blocks of molecular descriptors computed with Alvadesc

Regression algorithm	Descriptor set	Feature selection method	R2 (simple CV)	R2 (external data set)	RMSE (external data set)	R2 (nested CV)	CCC (nested CV) (95% CI)	RMSE (nested CV)
Linear regression	Set4	“carscore”	0.850	0.687	0.950	NA	NA	NA
Linear regression	Set4	Boruta	0.712	0.695	0.939	NA	NA	NA
Linear regression	Set4	Gaselect	0.798	0.692	0.943	NA	NA	NA
Glmnet	Set4	“find_correlation”	0.621	0.612	1.058	NA	NA	NA
Glmnet	Set4	Boruta	0.602	0.620	1.047	NA	NA	NA
Glmnet	Set4	Gaselect	0.612	0.598	1.078	NA	NA	NA
Weighted k-Nearest Neighbor	Set4	“information_gain”	0.926	0.834	0.692	0.665	0.829 (0.760 - 0.880)	0.923

**Regression algorithm**	**Descriptor set**	**Feature selection method**	**R2 (simple CV)**	**R2 (external data set)**	**RMSE (external data set)**	**R2 (nested CV)**	**CCC (nested CV) (95% CI)**	**RMSE (nested CV)**
Weighted k-Nearest Neighbor	Set4	Boruta	0.900	0.816	0.729	0.692	0.844 (0.774 - 0.895)	0.860
Weighted k-Nearest Neighbor	Set4	Gaselect	0.852	0.755	0.842	0.622	0.801 (0.725 - 0.857)	0.963
Random forest (“ranger”)	Set4	“information_gain”	0.928	0.763	0.827	0.652	0.790 (0.729 –0.838)	0.948
Random forest (“ranger”)	Set4	Boruta	0.936	0.775	0.806	0.702	0.831 (0.776 - 0.874)	0.861
Random forest (“ranger”)	Set4	Gaselect	0.895	0.809	0.742	0.676	0.801 (0.738 - 0.850)	0.910
Support vector machines	Set4	“jmim”	0.826	0.759	0.835	0.637	0.768 (0.691 - 0.828)	0.975
Support vector machines	Set4	Boruta	0.871	0.824	0.714	0.581	0.768 (0.673 - 0.837)	0.995
Support vector machines	Set4	Gaselect	0.788	0.627	1.038	NA	NA	NA
XGboost	Set4	“jmim”	0.933	0.730	0.883	0.701	0.835 (0.765 - 0.885)	0.857
XGboost	Set4	Boruta	0.950	0.787	0.784	0.717	0.847 (0.793 - 0.887)	0.834
XGboost	Set4	Gaselect	0.959	0.781	0.796	0.654	0.816 (0.741 - 0.871)	0.946
BART	Set4	“jmim”	0.953	0.749	0.852	0.683	0.835 (0.781 –0.876)	0.890
BART	Set4	Boruta	0.975	0.799	0.761	0.715	0.861 (0.820 –0.893)	0.845

Regression algorithm	Descriptor set	Feature selection method	R2 (simple CV)	R2 (external data set)	RMSE (external data set)	R2 (nested CV)	CCC (nested CV) (95% CI)	RMSE (nested CV)
BART	Set4	Gaselect	0.915	0.745	0.858	0.699	0.837 (0.783 - 0.879)	0.847
Multivariate Adaptive Regression Splines (“earth”)	Set4	“mim”	0.627	0.543	1.149	NA	NA	NA
Multivariate Adaptive Regression Splines (“earth”)	Set4	Boruta	0.698	0.652	1.003	NA	NA	NA
Multivariate Adaptive Regression Splines (“earth”)	Set4	Gaselect	0.671	0.710	0.980	NA	NA	NA
Gradient boosting machine (“GBM”)	Set4	“mim”	0.961	0.732	0.881	0.630	0.803 (0.734 - 0.856)	0.959
Gradient boosting machine (“GBM”)	Set4	Boruta	0.984	0.730	0.883	0.668	0.827 (0.779 - 0.865)	0.932
Gradient boosting machine (“GBM”)	Set4	Gaselect	0.828	0.658	0.994	NA	NA	NA
Regularized suport vector regression (“LiblineaR”)	Set4	“mim”	0.773	0.597	1.078	NA	NA	NA
Regularized suport vector	Set4	Boruta	0.711	0.694	0.940	NA	NA	NA

**Regression algorithm**	**Descriptor set**	**Feature selection method**	**R2 (simple CV)**	**R2 (external data set)**	**RMSE (external data set)**	**R2 (nested CV)**	**CCC (nested CV) (95% CI)**	**RMSE (nested CV)**
regression (“LiblineaR”)								
Regularized suport vector regression (“LiblineaR”)	Set4	Gaselect	0.828	0.632	1.030	NA	NA	NA
Conditional Random Forest	Set4	“mim”	0.868	0.746	0.856	0.669	0.789 (0.727 - 0.839)	0.926
Conditional Random Forest	Set4	Boruta	0.770	0.726	0.889	0.699	0.828 (0.773 - 0.871)	0.868
Conditional Random Forest	Set4	Gaselect	0.692	0.537	1.157	0.654	0.778 (0.721 –0.825)	0.943
Conditional inference trees	Set4	“mim”	0.772	0.702	0.928	0.366	0.647 (0.541 - 0.733)	1.284
Conditional inference trees	Set4	Boruta	0.789	0.786	0.787	0.541	0.766 (0.683 - 0.830)	1.074
Conditional inference trees	Set4	Gaselect	0.674	0.264	1.458	0.549	0.750 (0.674 - 0.810)	1.089
Rule- and instance-cased regression	Set4	“jmim”	0.798	0.671	0.975	NA	NA	NA
Rule- and instance-cased regression	Set4	Boruta	0.823	0.749	0.852	0.628	0.793 (0.720 –0.848)	0.964
Rule- and instance-cased regression	Set4	Gaselect	0.782	0.721	0.897	0.710	0.845 (0.795 - 0.883)	0.856

Regression algorithm	Descriptor set	Feature selection method	R2 (simple CV)	R2 (external data set)	RMSE (external data set)	R2 (nested CV)	CCC (nested CV) (95% CI)	RMSE (nested CV)
k-Nearest Neighbor	Set4	“jmim”	0.844	0.742	0.862	0.605	0.794 (0.717 –0.852)	1.009
k-Nearest Neighbor	Set4	Boruta	0.900	0.669	0.978	NA	NA	NA
k-Nearest Neighbor	Set4	Gaselect	0.850	0.687	0.950	NA	NA	NA
M5 rules	Set4	“mim”	0.783	0.664	0.985	NA	NA	NA
M5 rules	Set4	Boruta	0.774	0.704	0.924	0.409	0.718 (0.543 –0.833)	1.180
M5 rules	Set4	Gaselect	0.788	0.613	1.057	NA	NA	NA

Table S6. Performance of different models built with different regression algorithms and feature selection filters, using different blocks of molecular descriptors computed with Alvadesc

Regression algorithm	Descriptor set	Feature selection method	R2 (simple CV)	R2 (external data set)	RMSE (external data set)	R2 (nested CV)	CCC (nested CV) (95% CI)	RMSE (nested CV)
Linear regression	Set5	“correlation”	0.703	0.589	1.089	NA	NA	NA
Linear regression	Set5	Boruta	0.636	0.600	1.075	NA	NA	NA
Linear regression	Set5	Gaselect	0.751	0.565	1.121	NA	NA	NA
Glmnet	Set5	“information_gain”	0.633	0.643	1.016	NA	NA	NA
Glmnet	Set5	Boruta	0.621	0.616	1.053	NA	NA	NA
Glmnet	Set5	Gaselect	0.684	0.648	1.008	NA	NA	NA
Weighted k-Nearest Neighbor	Set5	“information_gain”	0.921	0.786	0.786 (sic!)	0.494	0.756 (0.681-0.815)	1.094
Weighted k-Nearest Neighbor	Set5	Boruta	0.918	0.790	0.779	0.628	0.826 (0.767 – 0.871)	0.939

Regression algorithm	Descriptor set	Feature selection method	R2 (simple CV)	R2 (external data set)	RMSE (external data set)	R2 (nested CV)	CCC (nested CV) (95% CI)	RMSE (nested CV)
Weighted k-Nearest Neighbor	Set5	Gaselect	0.895	0.723	0.894	0.639	0.826 (0.757 –0.877)	0.923
Random forest ("ranger")	Set5	"jmi"	0.887	0.758	0.837	0.625	0.779 (0.710 - 0.833)	0.973
Random forest ("ranger")	Set5	Boruta	0.922	0.806	0.748	0.654	0.803 (0.742 - 0.851)	0.927
Random forest ("ranger")	Set5	Gaselect	0.919	0.757	0.838	0.670	0.816 (0.759 - 0.860)	0.900
Support vector machines	Set5	"jmim"	0.802	0.706	0.922	0.586	0.748 (0.671 - 0.809)	1.029
Support vector machines	Set5	Boruta	0.828	0.724	0.893	0.656	0.812 (0.751 - 0.859)	0.916
Support vector machines	Set5	Gaselect	0.736	0.583	1.097	NA	NA	NA
XGboost	Set5	"jmim"	0.887	0.767	0.820	0.567	0.765 (0.685 - 0.827)	1.047
XGboost	Set5	Boruta	0.997	0.777	0.802	0.645	0.823 (0.762 - 0.869)	0.944
XGboost	Set5	Gaselect	0.911	0.746	0.857	0.654	0.826 (0.750 - 0.881)	0.897
BART	Set5	"mim"	0.894	0.661	0.990	0.584	0.789 (0.715 - 0.846)	0.995
BART	Set5	Boruta	0.919	0.793	0.774	0.656	0.812 (0.751 - 0.859)	0.916
BART	Set5	Gaselect	0.825	0.675	0.969	0.667	0.828 (0.769 - 0.873)	0.909

**Regression algorithm**	**Descriptor set**	**Feature selection method**	**R2 (simple CV)**	**R2 (external data set)**	**RMSE (external data set)**	**R2 (nested CV)**	**CCC (nested CV) (95% CI)**	**RMSE (nested CV)**
Multivariate Adaptive Regression Splines (“earth”)	Set5	“jmim”	0.552	0.610	1.061	NA	NA	NA
Multivariate Adaptive Regression Splines (“earth”)	Set5	Boruta	0.518	0.492	1.211	NA	NA	NA
Multivariate Adaptive Regression Splines (“earth”)	Set5	Gaselect	0.543	0.560	1.127	NA	NA	NA
Gradient boosting machine (“GBM”)	Set5	“mim”	0.915	0.778	0.801	0.499	0.755 (0.679 - 0.816)	1.068
Gradient boosting machine (“GBM”)	Set5	Boruta	0.886	0.770	0.815	0.537	0.778 (0.708 –0.832)	1.032
Gradient boosting machine (“GBM”)	Set5	Gaselect	0.660	0.571	1.113	NA	NA	NA
Regularized suport vector regression (“LiblineaR”)	Set5	“jmim”	0.732	0.544	1.147	NA	NA	NA
Regularized suport vector regression (“LiblineaR”)	Set5	Boruta	0.686	0.519	1.179	NA	NA	NA
Regularized suport	Set5	Gaselect	0.749	0.516	1.182	NA	NA	NA

Regression algorithm	Descriptor set	Feature selection method	R2 (simple CV)	R2 (external data set)	RMSE (external data set)	R2 (nested CV)	CCC (nested CV) (95% CI)	RMSE (nested CV)
vector regression (“LiblineaR”)								
Conditional Random Forest	Set5	“mim”	0.793	0.765	0.824	0.669	0.789 (0.727 - 0.839)	0.926
Conditional Random Forest	Set5	Boruta	0.623	0.635	1.027	NA	NA	NA
Conditional Random Forest	Set5	Gaselect	0.662	0.644	1.013	NA	NA	NA
Conditional inference trees	Set5	“disr”	0.725	0.552	1.138	NA	NA	NA
Conditional inference trees	Set5	Boruta	0.792	0.691	0.944	NA	NA	NA
Conditional inference trees	Set5	Gaselect	0.618	0.563	1.123	NA	NA	NA
Rule- and instance-cased regression	Set5	“jmim”	0.800	0.687	0.951	NA	NA	NA
Rule- and instance-cased regression	Set5	Boruta	0.904	0.771	0.813	0.470	0.748 (0.510 - 0.879)	1.089
Rule- and instance-cased regression	Set5	Gaselect	0.720	0.597	1.078	NA	NA	NA
k-Nearest Neighbor	Set5	“disr”	0.889	0.797	0.766	NA	NA	NA
k-Nearest Neighbor	Set5	Boruta	0.963	0.743	0.862	0.628	0.826 (0.767 –0.871)	0.939

**Regression algorithm**	**Descriptor set**	**Feature selection method**	**R2 (simple CV)**	**R2 (external data set)**	**RMSE (external data set)**	**R2 (nested CV)**	**CCC (nested CV) (95% CI)**	**RMSE (nested CV)**
k-Nearest Neighbor	Set5	Gaselect	0.858	0.726	0.890	0.639	0.826 (0.757 –0.877)	0.923
M5 rules	Set5	“jmim”	0.679	0.622	1.044	NA	NA	NA
M5 rules	Set5	Boruta	0.719	0.692	0.943	NA	NA	NA
M5 rules	Set5	Gaselect	0.723	0.593	1.084	NA	NA	NA
