# Supplementary material for: QSAR Regression Models for Predicting HMG-CoA Reductase Inhibition
Source: Pharmaceuticals (Basel). 2024 Oct 30;17(11):1448. doi: 10.3390/ph17111448 (PMC11597356; doi:10.3390/ph17111448)
Supplement: Supplementary file 1 [file pharmaceuticals-17-01448-s001.zip › Tables S7-S9.pdf]

Table S7. Key Descriptors Utilized in the Regression Model Constructed using the Set 4 descriptors (functional group counts, atom-centered fragments, atom-type E-state indices, and pharmacophore descriptors) (model no. 15 in Table 2). The model employed KKN as the regression algorithm with 'Boruta' as a feature selection method.

**Descriptor**	**Correlated Descriptors**	**Correlation coefficient(s)**	**Activity Relationship**
SaaaC (Sum of aaaC E-states, i.e. E-states for aromatic carbon atoms that have no hydrogen atoms attached and are connected to three other aromatic atoms)	NaaaC (Number of atoms of type aaaC)	r=0.98	Lower values → higher activity
C-033 (R–CH..X)	None	N/A	Higher values → higher activity
nCconj (number of non-aromatic conjugated C(sp2))	None	N/A	Higher values → higher activity
C-034 (R–CR..X)	nPyrroles (number of pyrrole rings), N-073 (Ar2NH / Ar3N / Ar2N-Al / R..N..R), SaasN (sum of aasN E-states), NaasN (number of atoms of type aasN)	r=0.89 – 0.90	Higher values → very slightly higher activity
SaaCH (Sum of aaCH E-states)	nBM (number of multiple bonds)<br>nAB (number of aromatic bonds)<br>nCsp2 (number of sp2 hybridized Carbon atoms)<br>ARR (aromatic ratio)<br>piPC04 (molecular multiple path count of order 4)<br>piPC05 (molecular multiple path count of order 5)<br>P_VSA_e_2 (P_VSA-like on Sanderson electronegativity, bin 2)<br>P_VSA_i_2 (P_VSA-like on ionization potential, bin 2)<br>P_VSA_s_4 (P_VSA-like on I-state, bin 4)<br>VSA_ppp_con (P_VSA-like on potential pharmacophore points, conjugated atoms)<br>P_VSA_ppp_cyc (P_VSA-like on potential pharmacophore points, atoms belonging to cycles)<br>Eta_betaP_A (eta pi and lone pair average VEM count)<br>Eta_D_beta_A (eta average measure of electronic features)<br>SpMAD_EA(bo) (spectral mean absolute deviation from edge adjacency mat. weighted by bond order)<br>nCar (number of aromatic C(sp2))<br>C-025 (R–CR–R)<br>Uc (unsaturation count)<br>Ui (unsaturation index)<br><br>P_VSA_MR_6 (P_VSA-like on Molar Refractivity, bin 6)<br>P_VSA_ppp_ar (P_VSA-like on potential pharmacophore points, aromatic atoms)<br>nCbH (number of unsubstituted benzene C(sp2))<br>C-024 (R–CH–R)	r > 0.80<br><br>r > 0.90	Higher values → slightly higher activity

Descriptor	Correlated Descriptors	Correlation coefficient(s)	Activity Relationship
	NaaCH (Number of atoms of type aaCH)		
C-001 (CH3R / CH4)	P_VSA_LogP_1 (P_VSA-like on LogP, bin 1)<br>NsCH3 (Number of atoms of type sCH3)<br><br>nCp (number of terminal primary C(sp3))<br>SsCH3 (Sum of sCH3 E-states)	r > 0.89<br><br><br>r > 0.90	Higher values → very slightly higher activity
nPyrroles (number of pyrrole rings)	C-034 (R–CR..X)<br>SaasN (Sum of aasN E-states)	r > 0.81	Higher values → very slightly higher activity
H-053 (H attached to C0(sp3) with 2X attached to next C)	None	NA	A flattened inverted U shape
SssCH2 (Sum of ssCH2 E-states)	X1Av (average valence connectivity index of order 1)<br>X3Av (average valence connectivity index of order 3)<br>X4Av (average valence connectivity index of order 4)<br>X5Av (average valence connectivity index of order 5)<br>ChiA_B(s) (average Randic-like index from Burden matrix weighted by I-State)<br>Eta_L_A (eta average local composite index)<br>C-002 (CH2R2)<br><br>SpPosA_B(e) (normalized spectral positive sum from Burden matrix weighted by Sanderson electronegativity)<br>SpPosA_B(i) (normalized spectral positive sum from Burden matrix weighted by ionization potential)<br>AVS_B(s) (average vertex sum from Burden matrix weighted by I-State	r > 0.80<br><br><br>r < - 0.80	Higher values → slightly lower activity

Table S8. Key Descriptors Utilized in the Regression Model Constructed using the Set 4 descriptors (functional group counts, atom-centered fragments, atom-type E-state indices, and pharmacophore descriptors) (model no. 16 in Table 2). The model employed BART as the regression algorithm and 'gaselect' as a feature selection method.

Descriptor	Correlated Descriptors	Correlation coefficient(s)	Activity Relationship
CATS2D_07_DL (CATS2D Donor-Lipophilic at lag 07)	CATS2D_02_DL (CATS2D Donor-Lipophilic at lag 02)<br>CATS2D_09_DL (CATS2D Donor-Lipophilic at lag 09)	r > 0.81	Higher values → higher activity
C-034 (R–CR..X)	nPyrroles (number of pyrrole rings), N-073 (Ar2NH / Ar3N / Ar2N-Al / R..N..R), SaasN (sum of aasN E-states), NaasN (number of atoms of type aasN)	r= 0.89 – 0.90	Higher values → higher activity
nPyrimidines (number of Pyrimidines)	nSO2N (number of sulfonamides (thio-/dithio-)<br>C-032 (X–CX–X)	r= 0.80 – 0.87	Higher values → higher activity

Descriptor	Correlated Descriptors	Correlation coefficient(s)	Activity Relationship
	N-071 (Ar-NAl2)<br>S-110 (R-SO2-R)<br>NddssS (number of atoms of type ddssS)<br>B01[N-S] (presence/absence of N – S at topological distance 1)<br>B01[O-S] (presence/absence of O – S at topological distance 1)<br>B03[N-S] (presence/absence of N – S at topological distance 3)<br>B09[O-S] (presence/absence of O – S at topological distance 9)<br>B09[S-F] (presence/absence of S – F at topological distance 9)<br>F01[N-S] (frequency of N – S at topological distance 1)<br>F01[O-S] (frequency of O – S at topological distance 1)<br>F03[N-S] (frequency of N – S at topological distance 3)<br>F09[O-S] (frequency of O – S at topological distance 9)<br>F09[S-F] (frequency of S – F at topological distance 9)		
CATS2D_07_DA (CATS2D Donor-Acceptor at lag 07)	None	N/A	Higher values → lower activity
C-033 (R–CH..X)	None	N/A	Higher values → higher activity
CATS2D_03_DL (CATS2D Donor-Lipophilic at lag 03)	TIE (E-state topological parameter)<br>BAC (Balaban centric index)<br>J_A (Balaban-like index from adjacency matrix)<br>J_X (Balaban-like index from chi matrix)<br>H_Dt (Harary-like index from detour matrix)<br>Wi_D/Dt (Wiener-like index from distance/detour matrix)<br>AVS_Dz(v) (average vertex sum from Barysz matrix weighted by van der Waals volume)<br>SpAbs_Dz(v) (graph energy from Barysz matrix weighted by van der Waals volume)<br>SpPos_Dz(v) (spectral positive sum from Barysz matrix weighted by van der Waals volume)<br>SpMax_Dz(v) (leading eigenvalue from Barysz matrix weighted by van der Waals volume)<br>SpAD_Dz(v) (spectral absolute deviation from Barysz matrix weighted by van der Waals volume)<br>J_B(m) (Balaban-like index from Burden matrix weighted by mass)<br>J_B(v) (Balaban-like index from Burden matrix weighted by van der Waals volume)<br>J_B(p) Balaban-like index from Burden matrix weighted by polarizability<br>J_B(i) (Balaban-like index from Burden matrix weighted by ionization potential)<br>ATSC5e (Centred Broto-Moreau autocorrelation of lag 5 weighted by Sanderson electronegativity)<br>ATSC6e (Centred Broto-Moreau autocorrelation of lag 6 weighted by Sanderson electronegativity)	r > 0.80	Higher values → lower activity

Descriptor	Correlated Descriptors	Correlation coefficient(s)	Activity Relationship
	ATSC5s (Centred Broto-Moreau autocorrelation of lag 5 weighted by I-state)<br>ATSC6s (Centred Broto-Moreau autocorrelation of lag 6 weighted by I-state)<br>ATSC7s (Centred Broto-Moreau autocorrelation of lag 7 weighted by I-state)<br>P_VSA_LogP_4 (P_VSA-like on LogP, bin 4)<br>P_VSA_MR_2 (P_VSA-like on Molar Refractivity, bin 2)<br>P_VSA_v_2 (P_VSA-like on van der Waals volume, bin 2)<br>P_VSA_p_2 (P_VSA-like on polarizability, bin 2)<br>nHDon (number of donor atoms for H-bonds (N and O))<br>H-050 (H attached to heteroatom)<br>SdO (Sum of dO E-states)<br>CATS2D_00_DD (CATS2D Donor-Donor at lag 00 (number of H bond donor atoms))<br>CATS2D_03_DD (CATS2D Donor-Donor at lag 03)<br>CATS2D_08_DD (CATS2D Donor-Donor at lag 08)<br>CATS2D_09_DD (CATS2D Donor-Donor at lag 09)<br>CATS2D_03_DA (CATS2D Donor-Acceptor at lag 03)<br>CATS2D_05_DA (CATS2D Donor-Acceptor at lag 05)<br>CATS2D_08_DA (CATS2D Donor-Acceptor at lag 08)<br>CATS2D_09_DA (CATS2D Donor-Acceptor at lag 09)<br>CATS2D_04_DL (CATS2D Donor-Lipophilic at lag 04)<br>F04[C-O] (Frequency of C – O at topological distance 4)<br>F08[O-O] (Frequency of O – O at topological distance 8)<br>Hy (hydrophilic factor)<br>TPSA(NO) (topological polar surface area using N,O polar contributions)<br>TPSA(Tot) (topological polar surface area using N,O,S,P polar contributions)<br>SAacc (surface area of acceptor atoms from P_VSA-like descriptors)		
SaaaC (Sum of aaaC E-states, i.e. E-states for aromatic carbon atoms that have no hydrogen atoms attached and are connected to three other aromatic atoms)	NaaaC (Number of atoms of type aaaC)	r=0.98	Lower values → higher activity
O-056 (alcohol)	P_VSA_MR_3 (P_VSA-like on Molar Refractivity, bin 3)<br>nOHs (number of secondary alcohols)<br>nROH (number of hydroxyl groups)	r > 0.96<br>r = 0.81	Higher values → lower activity
C-002 (CH2R2)	X3Av (average valence connectivity index of order 3)<br>X4Av (average valence connectivity index of order 4)<br>X5Av (average valence connectivity index of order 5)		Sawtooth-like curve (maximum

Descriptor	Correlated Descriptors	Correlation coefficient(s)	Activity Relationship
	ChiA_B(s) (average Randic-like index from Burden matrix weighted by I-State)<br>Eta_L_A (eta average local composite index)<br>SssCH2 (Sum of ssCH2 E-state)<br><br>nCs (number of total secondary C(sp3))<br>NssCH2 (Number of atoms of type ssCH2)	r > 0.80<br><br><br><br>r > 0.90	activity for lowest value)
C-006 (CH2RX)	None	N/A	Higher values → lower activity

Table S9. Key Descriptors Utilized in the Regression Model Constructed using the Set 4 descriptors (functional group counts, atom-centered fragments, atom-type E-state indices, and pharmacophore descriptors) (model no. 20 in Table 2). The model employed BART as the regression algorithm and 'Boruta' as a feature selection method.

Descriptor	Correlated Descriptors	Correlation coefficient(s)	Activity Relationship
nPyrroles (number of pyrrole rings)	C-034 (R–CR..X)<br>SaasN (Sum of aasN E-states)	r > 0.81	Higher values → very slightly higher activity
H-053 (H attached to C0(sp3) with 2X attached to next C)	None	NA	An inverted U shape
nCconj (number of non-aromatic conjugated C(sp2))	None	N/A	Higher values → higher activity (slight effect)
H-046 (H attached to C0(sp3) no X attached to next C)	H% (percentage of H atoms)<br>X0Av (average valence connectivity index of order 0)<br>Eta_L_A (eta average local composite index)<br><br>SpPosA_B(e) (normalized spectral positive sum from Burden matrix weighted by Sanderson electronegativity)<br>SpPosA_B(i) (normalized spectral positive sum from Burden matrix weighted by ionization potential)<br>Eta_beta_A (eta average VEM count)<br>Eta_FL_A (eta average local functionality index)	r > 0.81<br><br><br><br>r < -0.82	Sawtooth-like curve (maximum activity for lowest value)
SaaaC (Sum of aaaC E-states, i.e. E-states for aromatic carbon atoms that have no hydrogen atoms attached and are connected to three other aromatic atoms)	NaaaC (Number of atoms of type aaaC)	r=0.98	Sawtooth-like curve (maximum activity at minimum values)

**Descriptor**	**Correlated Descriptors**	**Correlation coefficient(s)**	**Activity Relationship**
CATS2D_06_AL (CATS2D Acceptor-Lipophilic at lag 06)	CATS2D_07_AL (CATS2D Acceptor-Lipophilic at lag 07)	r = 0.87	Higher values → lower activity
C-033 (R–CH..X)	None	N/A	Higher values → higher activity
CATS2D_04_AA (CATS2D Acceptor-Acceptor at lag 04)	F04[O-O] (Frequency of O – O at topological distance 4)	r=0.81	≥3 → Stronger activity
C-034 (R–CR..X)	nPyrroles (number of pyrrole rings), N-073 (Ar2NH / Ar3N / Ar2N-Al / R..N..R), SaasN (sum of aasN E-states), NaasN (number of atoms of type aasN)	R=0.89 – 0.90	Inverted U-shaped curve
nCrt (number of ring tertiary C)	nCt, C-003, SpMin1_Bh(s) (smallest eigenvalue n. 1 of Burden matrix weighted by I-state)	0.80 – 0.88	U-shaped curve
